# Clinical Walking Speed, Free-Living Walking Cadence, and All-Cause Mortality

**DOI:** 10.1001/jamanetworkopen.2026.40122

**Published:** 2026-10-23

**Authors:** Rayane Haddadj, Ian Meneghel Danilevicz, Alex V. Rowlands, Vincent T. van Hees, Benjamin Landré, Alexis Le Faucheur, Archana Singh-Manoux, Paul Jarle Mork, Séverine Sabia

**Affiliations:** 1 Department of Public Health and Nursing, Norwegian University of Science and Technology, Trondheim, Norway; 2 Centre for Research in Epidemiology and Statistics, Epidemiology of Ageing and Neurodegenerative Diseases, Université Paris Cité, Inserm U1153, National Research Institute for Agriculture, Food and Environment, Paris, France; 3 Assessment of Movement Behaviours Group, Diabetes Research Centre, College of Life Sciences, University of Leicester, Leicester, United Kingdom; 4 Leicester Diabetes Centre, Leicester General Hospital, University Hospitals of Leicester National Health Service Trust, Leicester, United Kingdom; 5 Alliance for Research in Exercise, Nutrition and Activity, School of Allied Health and Human Performance, Adelaide University, Adelaide, South Australia, Australia; 6 Accelting, Almere, the Netherlands; 7 University of Rennes, Laboratoire Mouvement, Sport, Santé, Rennes, France; 8 Faculty of Brain Sciences, University College London, London, United Kingdom

## Abstract

**Importance:**

Walking speed has been shown to be associated with mortality and is conventionally obtained from short walking tests in clinical settings. Free-living walking parameters such as walking cadence can be derived from accelerometers.

**Objective:**

To examine whether walking parameters measured in clinical and free-living settings are associated with comparable discriminative ability for all-cause mortality risk.

**Design, Setting, and Participants:**

This population-based cohort study, the Whitehall II accelerometer substudy in the UK, included a baseline from January 2012 to December 2013 and follow-up through August 19, 2024. The study included participants with valid accelerometer data, clinical walking speed, covariates, and electronic health records information. Analyses were performed between September 2025 and February 2026.

**Exposures:**

Clinical walking speed in meters per second was obtained from a 2.44-m walking test, and 2 free-living metrics of walking cadence in steps per minute (the median walking cadence and the 95th percentile walking cadence) were derived from 9 days of wrist-worn accelerometer data.

**Main Outcomes and Measures:**

All-cause mortality was ascertained from a British national mortality register. Cox proportional hazards regression models were used to estimate hazard ratios (HRs) for all-cause mortality. Discriminative ability was assessed using the Harrel concordance index (C index).

**Results:**

A total of 4067 participants (mean [SD] age, 69.3 [5.7] years; 3024 males [74.4%]) were included in the analysis. The median (IQR) follow-up was 11.8 (11.5-12.1) years. In models containing mortality risk factors (sociodemographic, behavioral, and health-related factors), higher clinical walking speed (HR, 0.77 [95% CI, 0.71-0.84]), median walking cadence (HR, 0.83 [95% CI, 0.77-0.90]), and 95th percentile walking cadence (HR, 0.82 [95% CI, 0.75-0.89]) were associated with lower all-cause mortality risk (per 1 higher SD) and with a similar discriminative ability (clinical walking speed: C index, 0.747 [95% CI, 0.730-0.764]; median walking cadence: C index, 0.744 [95% CI, 0.726-0.761]; and 95th percentile walking cadence: C index, 0.745 [95% CI, 0.727-0.762]). Only clinical walking speed was associated with an improved discriminative ability of a model including mortality risk factors.

**Conclusions and Relevance:**

In this cohort study, clinical walking speed and free-living walking cadence were both associated with all-cause mortality risk and had a similar discriminative ability for all-cause mortality. These results suggest that both measures can be used in population-based studies, and the choice between the 2 methods may depend on research objectives, implementation constraints, and the strengths of each method.

## Key Points

**Question:**

Are clinical walking speed and free-living walking cadence associated with a similar discriminative ability for all-cause mortality risk?

**Findings:**

This cohort study of 4067 participants found that clinical walking speed and free-living walking cadence were both associated with all-cause mortality and had a similar discriminative ability.

**Meaning:**

These findings suggest that both measures can be used in population-based studies, and the choice between the 2 methods may depend on research objectives, implementation constraints, and the strengths of each method.

## Teaser

This cohort study examines the association of clinical walking speed and free-living walking cadence with all-cause mortality risk and compares their discriminative ability among a subset of participants in the Whitehall II study.

## Introduction

Walking speed is known to be associated with risk of incident disability,^1^ morbidity,^2^ and mortality,^3^ particularly in older adults. Walking speed is conventionally measured using standardized walking tests^4^ or self-report questionnaires.^5^ Such assessments provide only a snapshot and are not fully representative of walking speed in free-living conditions.^6,7^ Free-living walking parameters can represent a complementary or alternative, scalable assessment by offering a detailed overview of daily function and may improve the monitoring and prediction of health outcomes.^8^

Over the past few decades, the use of body-worn sensors in large-scale population-based cohort studies has enabled novel insights in the field of physical activity, sedentary behavior, and sleep epidemiology through the processing of multiday accelerometer recordings.^9,10,11,12^ Algorithms have been developed to derive multiple walking parameters, such as walking events,^13^ step count,^14^ and walking cadence,^15^ allowing examination of their association with health outcomes. Ascertainment of walking speed from accelerometers remains challenging, particularly using wrist-worn devices, but walking cadence—number of steps per unit of time when walking, a component of walking speed—can be derived and used as a proxy. Daily step count has a dose-response association with several health events, including mortality.^16,17,18^ Recent evidence also suggests an association between walking cadence and mortality risk, irrespective of step count.^17^

Walking parameters obtained from a clinical walking test and a free-living accelerometer recording can both be used to estimate the speed at which an individual usually walks. Yet studies suggest that these measures are only weakly correlated. In this study, we aimed to examine the association of walking speed obtained from a standardized walking test and walking cadence obtained from a free-living accelerometer recording with all-cause mortality and compare their discriminative ability.

## Methods

### Study Design

The Whitehall II study is an ongoing prospective cohort study that was established from August 1985 to April 1988, comprising 10 308 British civil servants who have undergone clinical examinations every 4 to 5 years. An accelerometer measurement was added to the January 2012 to December 2013 wave of data collection for participants seen at a London clinic and those living in the Southeastern regions of England, who underwent clinical examination at home.^19^ Written informed consent for participation was obtained at each contact. Research ethics approval was obtained from the University College London ethics committee at each wave. This study is reported in accordance with the Strengthening the Reporting of Observational Studies in Epidemiology (STROBE) reporting guideline for cohort studies.

### Clinical Walking Speed

Walking speed was measured using a standardized protocol at the 2012 to 2013 clinical examination.^20^ Over a clearly marked 2.44-m walking course, participants were asked to “Walk to the other end of the course at [their] usual walking pace, just as if [they] were walking down the street to go to the shops. Walk all the way past the other end of the tape before you stop.” The starting position was standing at the start of the course. A trained nurse walked behind the participant and stopped timing when the participant’s foot hit the floor after the end of the walking course. The test was repeated 3 times, and the mean of 3 trials (meters per second) was retained for the analysis.^21^ Use of a walking stick, if habitual, was allowed.

### Free-Living Walking Parameters

At the 2012 to 2013 clinical examination, participants were asked to wear a GENEActiv accelerometer (ActivInsights Ltd) for 9 consecutive days on their nondominant wrist.^22^ Raw data, recorded at 85.7 Hz, were processed using the OxWearables/stepcount software, version 3.13.0 with 1 minor custom modification entailing the removal of the first incomplete minute in the raw data.^23^ The software performs data resampling, calibration to the local gravity, and nonwear time detection. Subsequently, walking was detected over 10-second windows using a machine-learning model, and step count was ascertained from every window classified as walking through detection of the local peaks on the vector magnitude. Participants with at least 1 valid day (≥16 hours of wear time with at least 1 step over the day) were included in the analysis.

Walking cadence, expressed as the number of steps per minute of walking, was calculated as the number of steps recorded in each 10-second window detected as walking multiplied by 6. For this study, 2 metrics were derived across valid days: the median and the 95th percentile of walking cadence across all detected 10-second windows of walking. The mean daily step count across valid days was also computed.

### Mortality and Covariates

All-cause mortality was ascertained from linked records from the British national mortality register (National Health Services Central Registry) with follow-up through August 19, 2024. Covariates were selected to represent known mortality risk factors and included sociodemographic (age, sex, educational level, self-reported race and ethnicity, and marital or cohabitation status), behavioral (smoking status, alcohol consumption, and fruit and vegetable consumption), and health-related (body mass index [BMI; calculated as weight in kilograms divided by height in meters squared], hypertension, hyperlipidemia, number of activities of daily living limitations, number of instrumental activities of daily living limitations, and number of chronic diseases among 10 diseases) factors. Self-reported race and ethnicity categories included minoritized race and ethnicity (Black, South Asian, and other) and White. Self-reported race and ethnicity were collected to account for and enable analysis of health disparities across demographic groups. Details are provided in the eMethods in Supplement 1.

### Statistical Analysis

Data analyses were performed between September 2025 and February 2026. We first calculated the correlation between clinical walking speed and the free-living walking parameters using the Pearson correlation coefficient. We then examined the association of clinical walking speed and free-living walking cadence metrics with all-cause mortality using hazard ratios (HRs) and 95% CIs derived from Cox proportional hazards regression models. The proportional hazards assumption was assessed using Schoenfeld residual plots, and no violations were observed. Model 1 was unadjusted. Model 2 was adjusted for sociodemographic factors, model 3 was adjusted further for behavioral factors, and model 4 was additionally adjusted for health-related factors. The models’ discriminative ability was assessed using the Harrel concordance index (C index), and associated 95% CIs were estimated using the delta-*U* estimate of the SE.^24^ *P* values for the difference in the C index were calculated using the delta-*U* test. Given the known sex differences in walking parameters and mortality risk, these analyses were repeated in female and male participants separately to check for consistency and interaction with sex. The calibration, for agreement between observed and predicted all-cause mortality risk with an 11-year prediction horizon of the different walking parameters, was tested using the Greenwood Nam-D’Agostino test.

Sensitivity analyses were designed to assess the robustness of our findings. First, to assess the potential impact of missing accelerometer data, we examined the association between clinical walking speed and mortality in all participants with available walking speed measures. Second, the main analysis was repeated by subgroups based on age (median split), BMI (<25 vs ≥25), and prevalent chronic disease in 2012 to 2013 (0 vs ≥1). *P* values for the difference in the C index by stratification group were calculated using the delta-*U* test. Third, we excluded patients with all-cause mortality who died within the first 2 years of follow-up. Fourth, we used the clinical walking speed obtained from the first walking test, and we repeated the analyses among participants with at least 7 valid days of accelerometry. Fifth, we used walking cadence metrics derived from: (1) walking bouts lasting 30 seconds or more and 60 seconds or more, (2) purposeful walking, defined as 40 steps or more per minute over a 10-second window,^25^ and (3) the first midnight-to-midnight recording period.

We conducted 2 post hoc analyses to investigate whether a combination of free-living walking parameters was associated with improvement in the models’ discriminative ability. In these analyses, we also considered daily step count; while it does not measure walking speed or cadence, it is a proxy of walking volume and is associated with mortality.^16,17,18^ First, for each level of adjustment, we compared the C index of models including clinical walking speed and that of models including various combinations of free-living walking parameters (median walking cadence, 95th percentile walking cadence, and daily step count). Second, we compared the C index of models including clinical walking speed only with those of models including clinical walking speed and 1 or more free-living walking parameters.

Analyses were conducted using R, version 4.5.2 (R Foundation for Statistical Computing). Statistical significance was set at 2-sided *P* < .05. The code used for data cleaning and statistical analyses is available via the project repository.^26^

## Results

After excluding participants without valid accelerometer data, clinical walking speed measurement, covariates, and data from electronic health records, the study population was composed of 4067 participants (1043 females [25.6%] and 3024 males [74.4%]; 306 [7.5%] with minoritized race and ethnicity and 3761 [92.5%] White) (eFigure 1 in Supplement 1). As compared with the 6244 participants who participated to the 2012 to 2013 wave of data collection, this study sample was younger (mean [SD] age, 69.3 [5.7] vs 70.8 [6.1] years; *P* < .001), composed of more males (74.4% vs 62.0%; *P* < .001), and overall healthier (eTable 1 in Supplement 1). The study sample mean (SD) clinical walking speed was 1.2 (0.3) m/s. Over the mean (SD) 7.9 (1.0) valid days of accelerometer recording, participants had a mean (SD) median walking cadence of 75.3 (9.9) and 95th percentile walking cadence of 112.2 (9.7) steps/min.

The correlations of clinical walking speed with free-living walking parameters were weak (*r* = 0.28 for median walking cadence and *r* = 0.27 for 95th percentile walking cadence) (eFigure 2 in Supplement 1). Median walking cadence and 95th percentile walking cadence were moderately correlated with each other (*r* = 0.55) and with daily step count (*r* = 0.47 for median walking cadence and *r* = 0.38 for 95th percentile walking cadence). These correlations appeared stronger in females compared with males (eFigures 3 and 4 in Supplement 1).

During a median (IQR) follow-up of 11.8 (11.5-12.1) years, 739 all-cause mortality deaths (18.2%) were recorded. Characteristics of the study sample in 2012 to 2013 overall and by sex are shown in Table 1. Females compared with males had a higher mean (SD) 95th percentile walking cadence (113.9 [9.5] vs 111.7 [9.7] steps/min) and lower clinical walking speed (1.0 [0.3] vs 1.2 [0.2]) and daily step count (9077 [3915] vs 9678 [3732]) (all *P* < .001). Descriptive statistics of the additional walking parameters’ metrics are shown in eTable 2 in Supplement 1.

**Table 1.** Sample Characteristics in 2012 to 2013 in the Total Study Population and by Sex in the Whitehall II Accelerometer Substudy^a^

Characteristic	Overall (N = 4067)	Sex		*P* value
		Female (n = 1043)	Male (n = 3024)	
**Sociodemographic factors**				
Age, mean (SD), y	69.3 (5.7)	69.3 (5.7)	69.3 (5.6)	.99
Educational level				
No academic qualifications	373 (9.2)	181 (17.4)	192 (6.3)	<.001
Lower secondary school	1287 (31.6)	365 (35.0)	922 (30.5)	
Higher secondary school	1135 (27.9)	254 (24.4)	881 (29.1)	
University	942 (23.2)	189 (18.1)	753 (24.9)	
Higher degree	330 (8.1)	54 (5.2)	276 (9.1)	
Married	3047 (74.9)	554 (53.1)	2493 (82.4)	<.001
Race and ethnicity				
Minoritized^b^	306 (7.5)	136 (13.0)	170 (5.6)	<.001
White	3761 (92.5)	907 (87.0)	2854 (94.4)	
**Behavioral factors**				
Smoking status				
Never smoker	1988 (48.9)	594 (57.0)	1394 (46.1)	<.001
Former smoker	1948 (47.9)	408 (39.1)	1540 (50.9)	
Current smoker	131 (3.2)	41 (3.9)	90 (3.0)	
Alcohol consumption				
No consumption	822 (20.2)	353 (33.8)	469 (15.5)	<.001
1-14 U/wk	2291 (56.3)	587 (56.3)	1704 (56.3)	
>14 U/wk	954 (23.5)	103 (9.9)	851 (28.1)	
Fruit and vegetable consumption				
Less than daily	839 (20.6)	183 (17.5)	656 (21.7)	<.001
Daily	867 (21.3)	170 (16.3)	697 (23.0)	
More than daily	2361 (58.1)	690 (66.2)	1671 (55.3)	
**Health-related factors**				
Body mass index, mean (SD)^c^	26.6 (4.3)	27.0 (5.3)	26.5 (3.9)	.006
Hypertension	2406 (59.2)	566 (54.3)	1840 (60.8)	<.001
Hyperlipidemia	2080 (51.1)	530 (50.8)	1550 (51.3)	.81
No. of limited ADLs, mean (SD)	0.2 (0.6)	0.2 (0.7)	0.2 (0.6)	.19
No. of limited IADLs, mean (SD)	0.2 (0.6)	0.3 (0.8)	0.2 (0.5)	<.001
No. of chronic diseases, mean (SD)	0.6 (0.8)	0.7 (0.9)	0.6 (0.8)	<.001
**Walking parameters, mean (SD)**				
Clinical walking speed, m/s	1.2 (0.3)	1.0 (0.3)	1.2 (0.2)	<.001
Median step cadence, steps/min	75.3 (9.9)	75.2 (10.4)	75.3 (9.8)	.64
Step cadence 95th percentile, steps/min	112.2 (9.7)	113.9 (9.5)	111.7 (9.7)	<.001
Daily step count	9524 (3788)	9077 (3915)	9678 (3732)	<.001
Days of accelerometry	7.9 (1.0)	7.9 (1.0)	7.9 (1.0)	.14

Abbreviations: ADLs, activities of daily living; IADLs, instrumental activities of daily living.

^a^ Data are reported as No. (%) unless otherwise indicated.

^b^ Minoritized race and ethnicity categories included Black, South Asian, and other race and ethnicity.

^c^ Calculated as weight in kilograms divided by height in meters squared.

Irrespective of the adjustment level, higher clinical walking speed, median walking cadence, and 95th percentile walking cadence were associated with lower all-cause mortality in the full sample as well as in sex-stratified analyses, with no evidence of interactions (Table 2). In fully adjusted models using the full sample, HRs per 1 higher SD of walking speed (HR, 0.77 [95% CI, 0.71-0.84]), median walking cadence (HR, 0.83 [95% CI, 0.77-0.90]), and 95th percentile walking cadence (HR, 0.82 [95% CI, 0.75-0.89]) were associated with lower all-cause mortality risk; these associations remained evident after further adjustment for daily step count (eTable 3 in Supplement 1). In the full sample, clinical walking speed had an HR per 0.1 m/s of 0.90 (95% CI, 0.87-0.93); 0.89 (95% CI, 0.82-0.96) in females, and 0.91 (95% CI, 0.87-0.94) in males. For median walking cadence, the full sample had an HR per 10 steps/min of 0.83 (95% CI, 0.76-0.90); 0.81 (95% CI, 0.68-0.97) in females, and 0.83 (95% CI, 0.76-0.91) in males. For 95th percentile walking cadence, the full sample had an HR per 10 steps/min of 0.81 (95% CI, 0.75-0.88); 0.80 (95% CI, 0.67-0.95) in females, and 0.82 (95% CI, 0.74-0.90) in males (see model 4 in eTable 4 in Supplement 1).

**Table 2.** Associations Between Walking Parameters and All-Cause Mortality in the Whitehall II Accelerometer Substudy

Model^b^	Walking parameters, HR per 1 higher SD (95% CI)^a^		
	Clinical walking speed	Free-living walking parameters	
		Median walking cadence	95th Percentile walking cadence
**Model 1^c^**			
Full sample	0.62 (0.58-0.67)	0.63 (0.59-0.68)	0.63 (0.59-0.69)
Females	0.56 (0.48-0.64)	0.62 (0.53-0.73)	0.62 (0.54-0.72)
Males	0.60 (0.55-0.65)	0.63 (0.58-0.69)	0.65 (0.59-0.71)
**Model 2^d^**			
Full sample	0.71 (0.65-0.77)	0.78 (0.72-0.84)	0.77 (0.71-0.83)
Females	0.67 (0.56-0.80)	0.74 (0.62-0.88)	0.72 (0.61-0.85)
Males	0.73 (0.66-0.79)	0.79 (0.72-0.86)	0.78 (0.71-0.86)
**Model 3^e^**			
Full sample	0.72 (0.66-0.78)	0.79 (0.73-0.86)	0.78 (0.72-0.84)
Females	0.69 (0.58-0.82)	0.76 (0.63-0.91)	0.74 (0.63-0.87)
Males	0.73 (0.67-0.80)	0.80 (0.73-0.87)	0.79 (0.72-0.87)
**Model 4^f^**			
Full sample	0.77 (0.71-0.84)	0.83 (0.77-0.90)	0.82 (0.75-0.89)
Females	0.75 (0.61-0.91)	0.80 (0.67-0.97)	0.81 (0.68-0.95)
Males	0.78 (0.71-0.86)	0.83 (0.76-0.91)	0.82 (0.75-0.90)

Abbreviation: HR, hazard ratio.

^a^ There was no difference between sexes in the association between walking parameters and all-cause mortality.

^b^ Full sample: n = 739 of 4067; females: n = 155 of 1043; and males: n = 584 of 3024.

^c^ Unadjusted.

^d^ Adjusted for sex (full sample model only), age, educational level, marital status, and race and ethnicity.

^e^ Model 2 and adjusted for smoking status, alcohol consumption, and fruit and vegetable consumption.

^f^ Model 2 and model 3 and adjusted for body mass index (calculated as weight in kilograms divided by height in meters squared), hypertension, hyperlipidemia, number of activities of daily living limitations, number of instrumental activities of daily living limitations, and number of chronic diseases.

Table 3 shows discriminative abilities of walking parameters. In the full sample, each walking parameter considered on its own had a modest discriminative ability (C index range, 0.613 [95% CI, 0.592-0.633] for 95th percentile walking cadence to 0.632 [95% CI, 0.612-0.652] for clinical walking speed). The model containing sociodemographic, behavioral, health-related factors and no walking parameter (model 4) had a C index of 0.742 (95% CI, 0.724-0.759). Adding clinical walking speed (C index, 0.747 [95% CI, 0.730-0.764]), median walking cadence (C index, 0.744 [95% CI, 0.726-0.761]), or 95th percentile walking cadence (C index, 0.745 [95% CI, 0.727-0.762]) was associated with a similar discriminative ability and with lower all-cause mortality risk. Overall, models including clinical walking speed had a significantly higher C index compared with models without walking parameters, irrespective of covariates in the model. Models, including 95th percentile walking cadence, had a significantly higher C index compared with models with sociodemographic and behavioral factors only. Clinical walking speed and the 2 free-living walking cadence metrics had a similar discriminative ability for all-cause mortality risk irrespective of other covariates. Greenwood-Nam-D’Agostino tests did not show statistically significant differences across walking parameters, suggesting a good calibration for all-cause mortality (eFigure 5 in Supplement 1).

**Table 3.** Discriminative Ability of Walking Parameters and All-Cause Mortality Risk in the Whitehall II Accelerometer Substudy

Model^a^	Walking parameters, C index (95% CI)			
	None	Clinical walking speed	Free-living walking parameters	
			Median walking cadence	95th Percentile walking cadence
**Model 1^b^**				
Full sample	NA	0.632 (0.612-0.653)	0.615 (0.595-0.636)	0.613 (0.592-0.633)
Females	NA	0.671 (0.629-0.712)	0.618 (0.574-0.662)^c^	0.625 (0.582-0.668)
Males	NA	0.638 (0.615-0.661)	0.615 (0.592-0.639)	0.607 (0.583-0.630)^c^
**Model 2^d^**				
Full sample	0.719 (0.701-0.737)	0.732 (0.714-0.749)^e^	0.725 (0.706-0.743)	0.726 (0.708-0.744)^e^
Females	0.741 (0.705-0.778)	0.760 (0.725-0.794)^e^	0.749 (0.711-0.786)	0.756 (0.720-0.792)^e^
Males	0.711 (0.690-0.732)	0.722 (0.701-0.742)^e^	0.715 (0.694-0.737)	0.716 (0.695-0.737)
**Model 3^f^**				
Full sample	0.725 (0.708-0.743)	0.736 (0.718-0.753)^e^	0.729 (0.711-0.747)	0.731 (0.713-0.749)^e^
Females	0.750 (0.714-0.787)	0.765 (0.731-0.800)^e^	0.757 (0.720-0.794)	0.763 (0.727-0.799)^e^
Males	0.717 (0.696-0.737)	0.726 (0.705-0.746)^e^	0.720 (0.700-0.741)	0.721 (0.700-0.742)
**Model 4^g^**				
Full sample	0.742 (0.724-0.759)	0.747 (0.730-0.764)^e^	0.744 (0.726-0.761)	0.745 (0.727-0.762)
Females	0.776 (0.740-0.811)	0.782 (0.747-0.816)	0.778 (0.742-0.814)	0.781 (0.745-0.816)
Males	0.732 (0.711-0.752)	0.737 (0.717-0.756)	0.733 (0.713-0.754)	0.734 (0.714-0.754)

Abbreviations: C index, Harrel concordance index; NA, not applicable.

^a^ Full sample: n = 739 of 4067; females: n = 155 of 1043; and males: n = 584 of 3024.

^b^ Unadjusted.

^c^ The C index is significantly different from the model containing clinical walking speed.

^d^ Adjusted for sex (full sample model only), age, educational level, marital status, and race and ethnicity.

^e^ The C index is significantly different from the model containing no walking parameter.

^f^ Model 2 and adjusted for smoking status, alcohol consumption, and fruit and vegetable consumption.

^g^ Model 2 and model 3 and adjusted for body mass index (calculated as weight in kilograms divided by height in meters squared), hypertension, hyperlipidemia, number of activities of daily living limitations, number of instrumental activities of daily living limitations, and number of chronic diseases.

In sex-stratified analyses, the C indices of models, including risk factors, were higher among females, although the difference reached significance only for model 4. The pattern of added discriminative ability was the same among females than for the full sample. In males, clinical walking speed appeared to have a better discriminative value than 95th percentile walking cadence in unadjusted models, and free-living walking cadence did not add to the discriminative ability compared with models including only risk factors (models 2-4).

### Sensitivity Analyses

In model 4, the HR for all-cause mortality per 1 SD of clinical walking speed in the 2012 to 2013 sample (N = 5492, irrespective of participation in the accelerometer substudy) (eTable 5 in Supplement 1) was 0.74 (95% CI, 0.68-0.79) m/s, and the C index was 0.757 (95% CI, 0.743-0.771). Analyses stratified by age, BMI, and prevalent chronic disease are shown in eFigures 6 to 8 in Supplement 1. In both model 1 and model 4, C indices were similar across subgroups. Sensitivity analyses restricting the study population to participants who did not die during the first 2 years of follow-up, participants with 7 or more valid days of accelerometer wear, defining walking cadence based on walking bouts lasting 30 seconds or more and 60 seconds or more, purposeful walking, or the first midnight-to-midnight recording period and clinical walking speed from the first trial of the walking test are presented in eFigures 9 to 15 in Supplement 1. HRs and discriminative ability were attenuated in models including median walking cadence derived from walking bouts lasting 60 seconds or more (eFigure 12 in Supplement 1). Results from all other analyses were consistent with the main analysis.

### Post Hoc Analyses

Models including several free-living walking parameters had significantly higher C indices than the model containing sociodemographic and behavioral factors only (model 3) but not when the model also included health-related factors (model 4) (Table 4). All models, including 1 or more walking parameters, performed similarly compared with the model including clinical walking speed only. Adding 95th percentile walking cadence and daily step count or all free-living walking parameters was associated with significant improvement in the discriminative ability of models containing clinical walking speed and sociodemographic and behavioral factors but not when the model also included health-related factors (Table 5). Clinical walking speed along with 95th percentile walking cadence had the highest C index (0.750 [95% CI, 0.733-0.767]), but this model did not perform significantly better than the model containing clinical walking speed and all covariates.

**Table 4.** Discriminative Ability of Clinical Walking Speed and Combinations of Free-Living Walking Parameters and All-Cause Mortality Risk in the Whitehall II Accelerometer Substudy (n = 739 of 4067)

Model	Walking parameters, C index (95% CI)^a^					
	None	Clinical walking speed	Combinations of free-living walking parameters			
			Median walking cadence and 95th percentile walking cadence	Median walking cadence and daily step count	95th Percentile walking cadence and daily step count	All free-living walking parameters
Model 1^b^	NA	0.632 (0.612-0.653)	0.627 (0.606-0.648)	0.635 (0.614-0.656)	0.637 (0.617-0.658)	0.642 (0.621-0.662)
Model 2^c^	0.719 (0.701-0.737)	0.732 (0.714-0.749)^d^	0.727 (0.709-0.745)^d^	0.728 (0.710-0.747)^d^	0.730 (0.712-0.748)^d^	0.730 (0.712-0.748)^d^
Model 3^e^	0.725 (0.708-0.743)	0.736 (0.718-0.753)^d^	0.732 (0.714-0.750)^d^	0.732 (0.714-0.750)^d^	0.734 (0.716-0.751)^d^	0.734 (0.716-0.752)^d^
Model 4^f^	0.742 (0.724-0.759)	0.747 (0.730-0.764)^d^	0.745 (0.728-0.763)	0.745 (0.727-0.762)	0.746 (0.728-0.763)	0.746 (0.728-0.763)

Abbreviations: C index, Harrel concordance index; NA, not applicable.

^a^ There were no significant differences in the C index between the models including different walking parameters.

^b^ Unadjusted.

^c^ Adjusted for sex, age, educational level, marital status, and race and ethnicity.

^d^ The C index is significantly different from the model containing no walking parameter.

^e^ Model 2 and adjusted for smoking status, alcohol consumption, and fruit and vegetable consumption.

^f^ Model 2 and model 3 and adjusted for body mass index (calculated as weight in kilograms divided by height in meters squared), hypertension, hyperlipidemia, number of activities of daily living limitations, number of instrumental activities of daily living limitations, and number of chronic diseases.

**Table 5.** Discriminative Ability of Models Combining Clinical Walking Speed and Free-Living Walking Parameters and All-Cause Mortality Risk in the Whitehall II Accelerometer Substudy (n = 739 of 4067)

Model	Walking parameters, C index (95% CI)							
	Clinical walking speed	Clinical walking speed + free-living walking parameters						
		+ Median walking cadence	+ 95th Percentile walking cadence	+ Daily step count	+ Median step cadence and 95th percentile walking cadence	+ Median step cadence and daily step count	+ 95th Percentile walking cadence and daily step count	+ All free-living walking parameters
Model 1^a^	0.632 (0.612-0.653)	0.654 (0.634-0.675)^b^	0.656 (0.636-0.675)^b^	0.649 (0.629-0.670)^b^	0.660 (0.640-0.680)^b^^,^^c^	0.659 (0.639-0.680)^b^^,^^d^	0.662 (0.642-0.682)^b^^,^^d^	0.664 (0.644-0.684)^b^^,^^c^^,^^d^^,^^e^
Model 2^f^	0.732 (0.714-0.749)	0.734 (0.716-0.752)	0.735 (0.718-0.753)^b^	0.735 (0.718-0.753)	0.736 (0.718-0.753)	0.736 (0.718-0.754)	0.737 (0.719-0.754)^b^	0.737 (0.720-0.755)^b^
Model 3^g^	0.736 (0.718-0.753)	0.738 (0.720-0.755)	0.739 (0.721-0.756)	0.738 (0.721-0.756)	0.739 (0.722-0.757)	0.739 (0.722-0.757)	0.740 (0.723-0.757)^b^	0.740 (0.723-0.758)^b^
Model 4^h^	0.747 (0.730-0.764)	0.749 (0.731-0.766)	0.750 (0.733-0.767)	0.749 (0.732-0.766)	0.750 (0.733-0.767)	0.750 (0.732-0.767)	0.750 (0.733-0.767)	0.750 (0.733-0.768)

Abbreviation: C index, Harrel concordance index.

^a^ Unadjusted.

^b^ The C index is significantly different from the model containing clinical walking speed as the only walking parameter.

^c^ The C index is significantly different from the model containing clinical walking speed and median step cadence as walking parameters.

^d^ The C index is significantly different from the model containing clinical walking speed, median step cadence, and step cadence 95th percentile as walking parameters.

^e^ The C index is significantly different from the model containing clinical walking speed, step cadence 95th percentile, and daily step count as walking parameters.

^f^ Adjusted for sex, age, educational level, marital status, and race and ethnicity.

^g^ Model 2 and adjusted for smoking, alcohol consumption, and fruit and vegetable consumption.

^h^ Model 2 and model 3 and adjusted for body mass index (calculated as weight in kilograms divided by height in meters squared), hypertension, hyperlipidemia, number of activities of daily living limitations, number of instrumental activities of daily living limitations, and number of chronic diseases.

## Discussion

This study found that clinical walking speed from a standardized walking test and free-living walking cadence from a wrist-worn accelerometer recording were associated with all-cause mortality in a large UK-based cohort study. Results on the discriminative ability of these models showed that clinical walking speed was associated with improved discriminative ability of models including known sociodemographic, behavioral, and health-related factors, while 95th percentile walking cadence was associated with improved discriminative ability of models containing sociodemographic and behavioral factors. Median walking cadence was not associated with models containing risk factors of mortality. There was no difference between sexes in the association between walking parameters and all-cause mortality. However, in fully adjusted models, C indices were higher in females compared with males, but there was no evidence of difference in discriminative ability between walking parameters. Walking parameters had a similar discriminative ability in the different subgroups defined by age, BMI, or prevalence of chronic diseases. Post hoc analyses suggested an association with improved discriminative ability when combining clinical walking speed and free-living walking parameters; however, this association was no longer significant when models also included health-related factors.

Prior studies have found that clinical walking speed is associated with mortality.^27,28,29,30^ A pooled analysis of 9 cohorts reported an HR of 0.88 (95% CI, 0.87-0.90) for a 0.1-m/s higher value of clinical walking speed,^3^ consistent with our findings when expressed on the same scale. We also found that the association and discriminative ability of walking speed were consistent whether we used the mean of 3 attempts or the first attempt only.

In the current study, walking cadence metrics were consistently associated with all-cause mortality, even after adjustment for daily step count, as has been reported elsewhere.^17,31^ Furthermore, estimates were largely similar for analysis restricted to (1) participants with 7 or more valid days of accelerometry, (2) walking bouts lasting 30 seconds or more and 60 seconds or more, (3) purposeful walking, and (4) the first midnight-to-midnight recording period, suggesting that a broad definition of walking cadence may adequately capture the association with all-cause mortality.

Previous studies have reported varying strengths of correlation between walking parameters measured in a laboratory or clinical setting and free-living assessments of walking parameters.^7,8,32,33,34^ The weak correlations observed between clinical walking speed and free-living walking cadence in this study reinforce the hypothesis of a disparity between walking parameters assessed in a standardized clinical setting and those derived from free-living condition. Furthermore, our post hoc analyses suggested an added value of combining clinical walking speed and free-living walking parameters in models including sociodemographic and behavioral factors but not when including health factors. This result extends previous findings by indicating that both clinical and free-living settings might convey relevant and complementary information for discriminating people at risk of mortality when measuring walking parameters. Future studies are needed to explore common and specific mechanisms that may relate clinical and free-living measurements of walking parameters to mortality and other health outcomes. Whether clinical or free-living measurements should be prioritized depends on the overall aim and ought to consider strengths of each method. Walking speed tests can be completed in a short time, are low cost, and require minimal data processing, whereas accelerometers allow multiday high-resolution recording and characterization of other exposures such as physical activity intensity, postures, and activity type. Accelerometers also allow for deriving walking cadence from datasets that were initially designed for other purposes and can benefit from advances in algorithm development. Furthermore, the increasing availability of commercial wearables could offer the opportunity to integrate free-living walking parameters in medical practice alongside other clinical measures, as well as providing real-time feedback during behavior change interventions.

We found that the correlations between clinical walking speed and free-living walking cadence variables were stronger in females compared with males, suggesting that different mechanisms may link clinical and free-living measurements across sexes. In addition, although the association between walking parameters and all-cause mortality did not differ significantly by sex, analyses showed that 95th percentile walking cadence added to the discriminative ability of models containing sociodemographic and behavioral factors in females but not in males. A previous study found that the addition of accelerometer-derived walking parameters were associated with the model’s improved discriminative ability in both sexes in the training cohort (UK Biobank), whereas this improvement was observed only among female participants in the validation cohort (National Health and Nutrition Examination Survey).^35^ Future research is needed to explore the possible sex-specific relationship between walking parameters and their discriminative ability for health outcomes.

### Strengths and Limitations

The strengths of our study include the use of a walking speed test and the accelerometer recording within the same study and at the same time point. Strengths also include the long follow-up period and sufficient number of deaths over the follow-up.

Our study also has limitations. First, there are no standardized methods for the derivation of walking cadence from accelerometers. We used 2 easily interpretable percentile-based summary metrics, but other summary metrics or complex accelerometer information may be relevant for health outcomes. Second, although walking cadence is a component of walking speed and correlates with walking intensity,^36,37^ the comparability between clinical walking speed and free-living walking cadence is limited, and they should not be considered interchangeable. Future studies including accelerometer-derived walking speed may allow for a more robust comparison. Third, the absence of a validation dataset limits our ability to assess the external validity of the models. Fourth, the availability of accelerometer data may be affected by place of residence, wear contraindications, nonconsent, and nonwear, resulting in a study sample healthier than the overall 2012 to 2013 sample. However, results remained consistent with the main analysis when including every participant with available clinical walking speed measurement and among participants with at least 1 chronic disease.

## Conclusions

In this population-based cohort study that included 4067 participants, clinical walking speed and free-living walking cadence were associated with a similar discriminative ability and all-cause mortality. Discriminative ability was associated with improvement when combining clinical walking speed and free-living walking parameters; however, this association was no longer significant when the models also included health-related factors. Whether clinical or free-living measurements should be prioritized must consider research aim and each method’s strengths. Future research is warranted to replicate our analysis in different settings and investigate common and specific mechanisms that may relate clinical and free-living measurements to health outcomes.

## Notes

Supplement 1. **eMethods.** Sociodemographic, Lifestyle, and Health-Related Factors in Whitehall II **eFigure 1.** Flowchart Diagram of Inclusion in the Study **eFigure 2.** Correlation Plot of Walking Parameters **eFigure 3. **Correlation Plot of Walking Parameters Among Female Participants **eFigure 4. **Correlation Plot of Walking Parameters Among Male Participants **eTable 1.** Descriptive Characteristics of Whitehall II Participants Comparing Included and Nonincluded in the Analysis **eTable 2.** Descriptive Statistics of Supplementary Walking Parameters Metrics for the Whole Study Sample and Stratified by Sex **eTable 3.** Association Between Free-Living Walking Cadence and All-Cause Mortality Adjusted for Daily Step Count (N cases/N total: Full Sample, 739/4067; Female, 155/1043; Male, 584/3024) **eTable 4.** Association Between the Walking Parameters and All-Cause Mortality in the Whitehall II Accelerometer Substudy for 0.1 m/s of Clinical Walking Speed and 10 Steps/Minute of Free-Living Walking Cadence **eTable 5.** Association Between Clinical Walking Speed and All-Cause Mortality Including All Participants With Valid Clinical Walking Speed Measurement (N cases/N total: 1079/5492) **eFigure 5.** Calibration of the Walking Parameters for the 11-Year Risk of All-Cause Mortality **eFigure 6.** Hazard Ratios (A) and Discriminative Ability (B) of Walking Parameters for All-Cause Mortality Risk by Age (N Cases/N Total: in Younger Group, age <68.4 Years, 166/2033; in Older Group, Age ≥68.4 Years, 573/2034) **eFigure 7.** Hazard Ratios (A) and Discriminative Ability (B) of Walking Parameters for All-Cause Mortality Risk by Body Mass Index (N Cases/N Total: in Those with BMI <25kg/m^2^, 281/1570; in Those with BMI ≥25 kg/m^2^, 458/2497) **eFigure 8.** Hazard Ratios (A) and Discriminative Ability (B) of Walking Parameters for All-Cause Mortality Risk by Prevalence of at Least 1 Chronic Disease at Baseline (N Cases/N Total: in Those With no Chronic Disease, 306/2278; in Those With at Least 1 Chronic Disease, 433/1789) **eFigure 9.** Hazard Ratios (A) and Discriminative Ability (B) of Walking Parameters for All-Cause Mortality Excluding Participants that Died During the First 2 Years of Follow-Up (N Cases/N Total = 691/4019) **eFigure 10.** Hazard Ratios (A) and Discriminative Ability (B) of Walking Parameters for All-Cause Mortality Risk Including Participants With ≥7 Valid Days of Accelerometer Wear (N Cases/N Total = 708/3905) **eFigure 11.** Hazard Ratios (A) and Discriminative Ability (B) of Walking Parameters for All-Cause Mortality Risk With Walking Cadence Derived From Walking Bouts ≥30 Seconds (N Cases/N Total = 739/4067) **eFigure 12.** Hazard Ratios (A) and Discriminative Ability (B) of Walking Parameters for All-Cause Mortality Risk With Walking Cadence Derived From Walking Bouts ≥60 Seconds (N Cases/N Total = 736/4061) **eFigure 13.** Hazard Ratios (A) and Discriminative Ability (B) of Walking Parameters for All-Cause Mortality Risk With Walking Cadence Derived From Purposeful Walking (≥40 Steps/Minute Over a 10-Second Window) (N cases/N total = 739/4067) **eFigure 14. **Hazard Ratios (A) and Discriminative Ability (B) of Walking Parameters for All-Cause Mortality Risk With Walking Cadence Derived From the First Complete 24-Hour (N Cases/N Total = 737/4064) **eFigure 15.** Hazard Ratios (A) and Discriminative Ability (B) of Walking Parameters for All-Cause Mortality With Clinical Walking Speed Derived From the First Trial of the Walking Test (N Cases/N Total = 739/4067)

Supplement 2.
Data Sharing Statement
