# Supplementary material for: Clinical Walking Speed, Free-Living Walking Cadence, and All-Cause Mortality
Source: JAMA Netw Open. 2026 Oct 23;9(10):e2640122. doi: 10.1001/jamanetworkopen.2026.40122 (PMC13695056; doi:10.1001/jamanetworkopen.2026.40122)
Supplement: Supplementary file 1 [file jamanetwopen-e2640122-s001.pdf]

Supplemental Online Content

Haddadj R, Danilevicz IM, Rowlands AV, et al. Clinical walking speed, free-living walking cadence, and all-cause mortality. *JAMA Netw Open*. 2026;9(10):e2640122.
doi:10.1001/jamanetworkopen.2026.40122

eMethods. Sociodemographic, Lifestyle, and Health-Related Factors in Whitehall II
eFigure 1. Flowchart Diagram of Inclusion in the Study
eFigure 2. Correlation Plot of Walking Parameters
eFigure 3. Correlation Plot of Walking Parameters Among Female Participants
eFigure 4. Correlation Plot of Walking Parameters Among Male Participants
eTable 1. Descriptive Characteristics of Whitehall II Participants Comparing Included and Nonincluded in the Analysis
eTable 2. Descriptive Statistics of Supplementary Walking Parameters Metrics for the Whole Study Sample and Stratified by Sex
eTable 3. Association Between Free-Living Walking Cadence and All-Cause Mortality Adjusted for Daily Step Count (N cases/N total: Full Sample, 739/4067; Female, 155/1043; Male, 584/3024)
eTable 4. Association Between the Walking Parameters and All-Cause Mortality in the Whitehall II Accelerometer Substudy for 0.1 m/s of Clinical Walking Speed and 10 Steps/Minute of Free-Living Walking Cadence
eTable 5. Association Between Clinical Walking Speed and All-Cause Mortality Including All Participants With Valid Clinical Walking Speed Measurement (N cases/N total: 1079/5492)
eFigure 5. Calibration of the Walking Parameters for the 11-Year Risk of All-Cause Mortality
eFigure 6. Hazard Ratios (A) and Discriminative Ability (B) of Walking Parameters for All-Cause Mortality Risk by Age (N Cases/N Total: in Younger Group, age <68.4 Years, 166/2033; in Older Group, Age ≥68.4 Years, 573/2034)
eFigure 7. Hazard Ratios (A) and Discriminative Ability (B) of Walking Parameters for All-Cause Mortality Risk by Body Mass Index (N Cases/N Total: in Those with BMI <25kg/m², 281/1570; in Those with BMI ≥25 kg/m², 458/2497)
eFigure 8. Hazard Ratios (A) and Discriminative Ability (B) of Walking Parameters for All-Cause Mortality Risk by Prevalence of at Least 1 Chronic Disease at Baseline (N Cases/N Total: in Those With no Chronic Disease, 306/2278; in Those With at Least 1 Chronic Disease, 433/1789)
eFigure 9. Hazard Ratios (A) and Discriminative Ability (B) of Walking Parameters for All-Cause Mortality Excluding Participants that Died During the First 2 Years of Follow-Up (N Cases/N Total = 691/4019)
eFigure 10. Hazard Ratios (A) and Discriminative Ability (B) of Walking Parameters for All-Cause Mortality Risk Including Participants With ≥7 Valid Days of Accelerometer Wear (N Cases/N Total = 708/3905)
eFigure 11. Hazard Ratios (A) and Discriminative Ability (B) of Walking Parameters for All-Cause Mortality Risk With Walking Cadence Derived From Walking Bouts ≥30 Seconds (N Cases/N Total = 739/4067)
eFigure 12. Hazard Ratios (A) and Discriminative Ability (B) of Walking Parameters for All-Cause Mortality Risk With Walking Cadence Derived From Walking Bouts ≥60 Seconds (N Cases/N Total = 736/4061)
eFigure 13. Hazard Ratios (A) and Discriminative Ability (B) of Walking Parameters for All-Cause Mortality Risk With Walking Cadence Derived From Purposeful Walking (≥40 Steps/Minute Over a 10-Second Window) (N cases/N total = 739/4067)
eFigure 14. Hazard Ratios (A) and Discriminative Ability (B) of Walking Parameters for All-Cause Mortality Risk With Walking Cadence Derived From the First Complete 24-Hour (N Cases/N Total = 737/4064)
eFigure 15. Hazard Ratios (A) and Discriminative Ability (B) of Walking Parameters for All-Cause Mortality With Clinical Walking Speed Derived From the First Trial of the Walking Test (N Cases/N Total = 739/4067)

This supplemental material has been provided by the authors to give readers additional information about their work.

© 2026 Haddadj R et al. *JAMA Network Open*.

eMethods. Sociodemographic, Lifestyle, and Health-Related Factors in Whitehall II

Data were drawn from questionnaires and clinical evaluations conducted in 2012-2013, as well as from electronic health records (Hospital Episode Statistics (HES), cancer registry, and the Mental Health Services Data Set).

Socio-demographic factors included age, sex, ethnicity (white, non-white), marital status (married/cohabitating, divorced/widowed/single), and education level (no academic qualifications, lower secondary school, higher secondary school, university higher degree; entered as an ordinal variable).

Behavioral factors were smoking status (current, past, never smoker), alcohol consumption (none, moderate (1-14 units/week), high (>14 units/week)), and fruit and vegetable consumption (less than daily, daily, twice or more daily).

Health-related factors included body mass index (kg/m²), hypertension (systolic/diastolic blood pressure ≥140/90 mmHg or use of antihypertensive drugs), hyperlipidemia (low-density lipoproteins >4.1 mmol/L or use of lipid-lowering drugs) assessed at the clinical examination, number of chronic diseases (among diabetes, congenital heart disease, stroke, heart failure, arthritis, cancer, Parkinson's disease, chronic obstructive pulmonary disease, and depression), number of limitations in activities of daily living (ADL, assessed by questions on limitations in the following activities: dressing, walking, bathing, eating, getting in bed, and using the toilet), and number of limitations in instrumental ADL (IADL, assessed by questions on limitations in the following activities: cooking, shopping for groceries, making telephone calls, taking medication, doing housework, and managing money).

eFigure 1. Flowchart Diagram of Inclusion in the Study

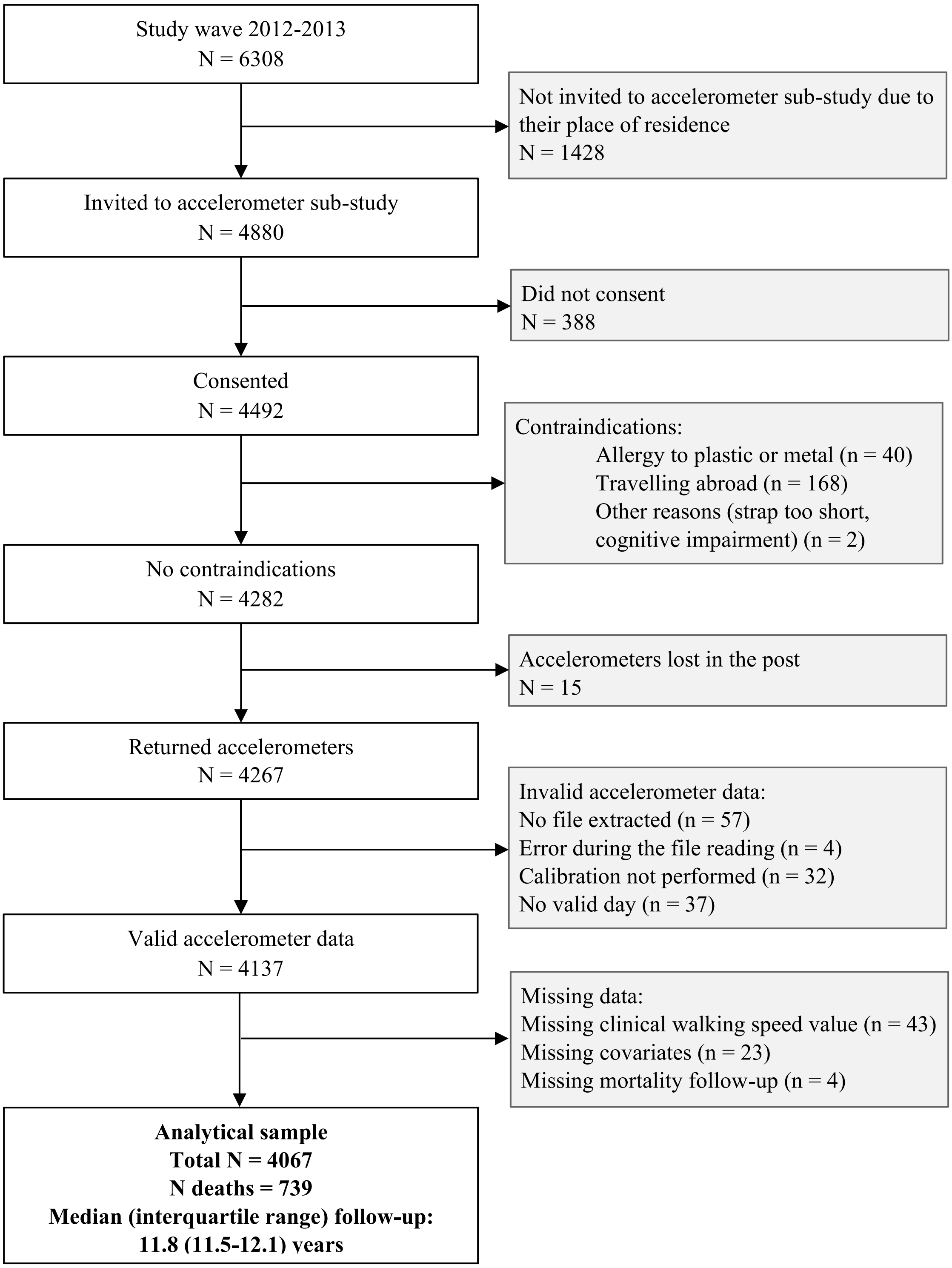

Valid day was defined as ≥16 hours of wear time with at least one step over the day

© 2026 Haddadj R et al. *JAMA Network Open*.

eFigure 2. Correlation Plot of Walking Parameters

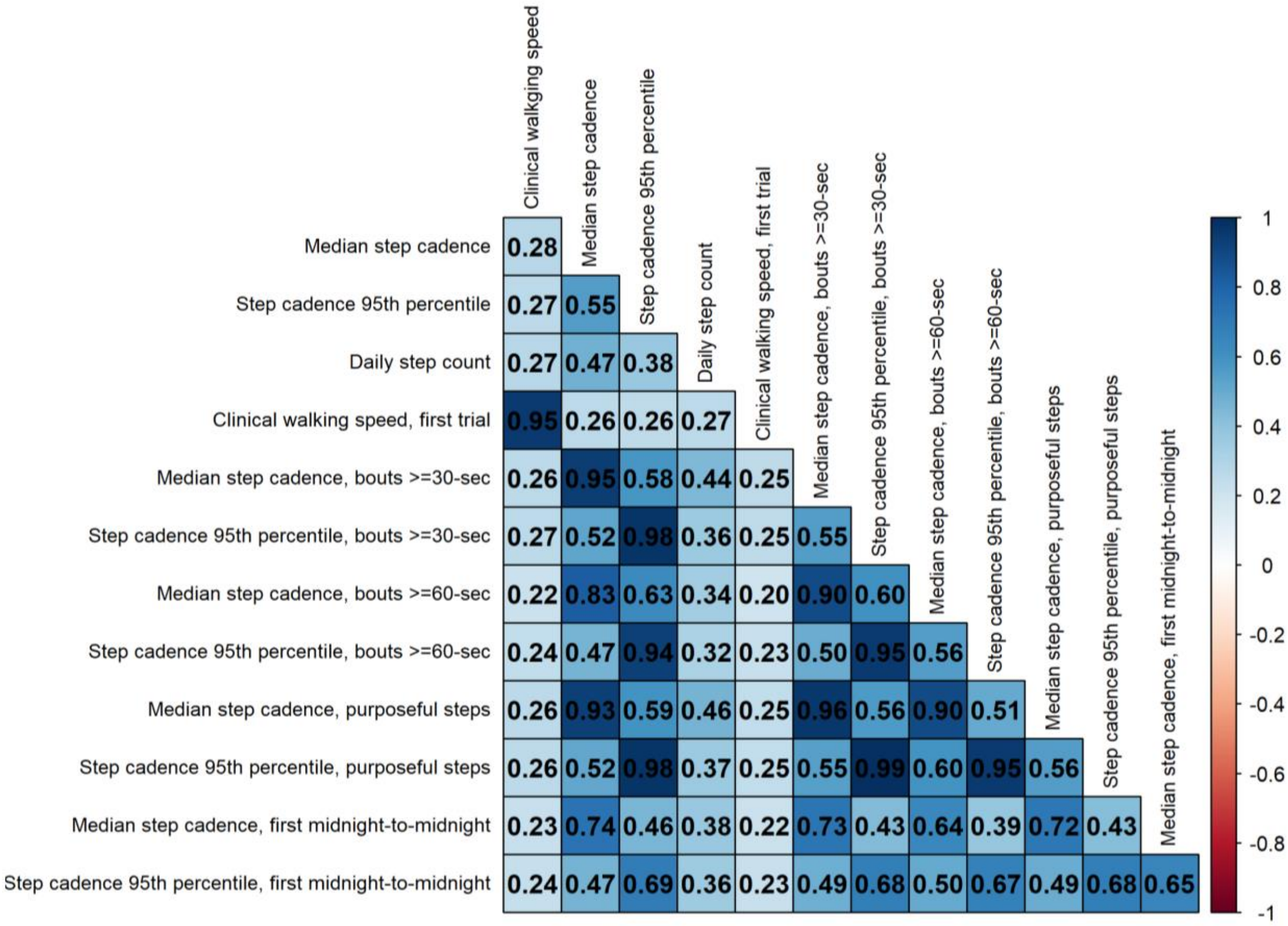

© 2026 Haddadj R et al. *JAMA Network Open*.

eFigure 3. Correlation Plot of Walking Parameters Among Female Participants

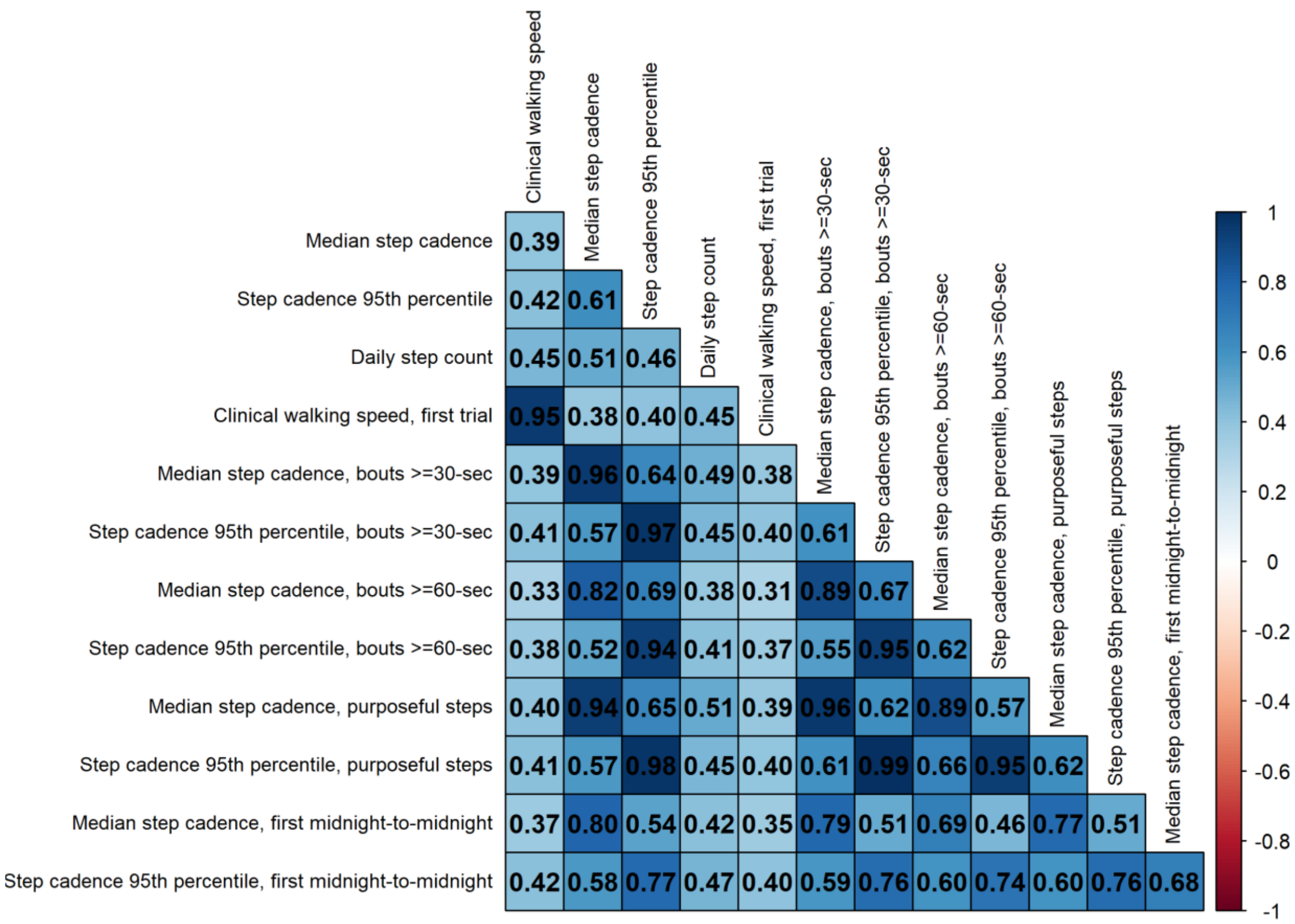

© 2026 Haddadj R et al. *JAMA Network Open*.

eFigure 4. Correlation Plot of Walking Parameters Among Male Participants

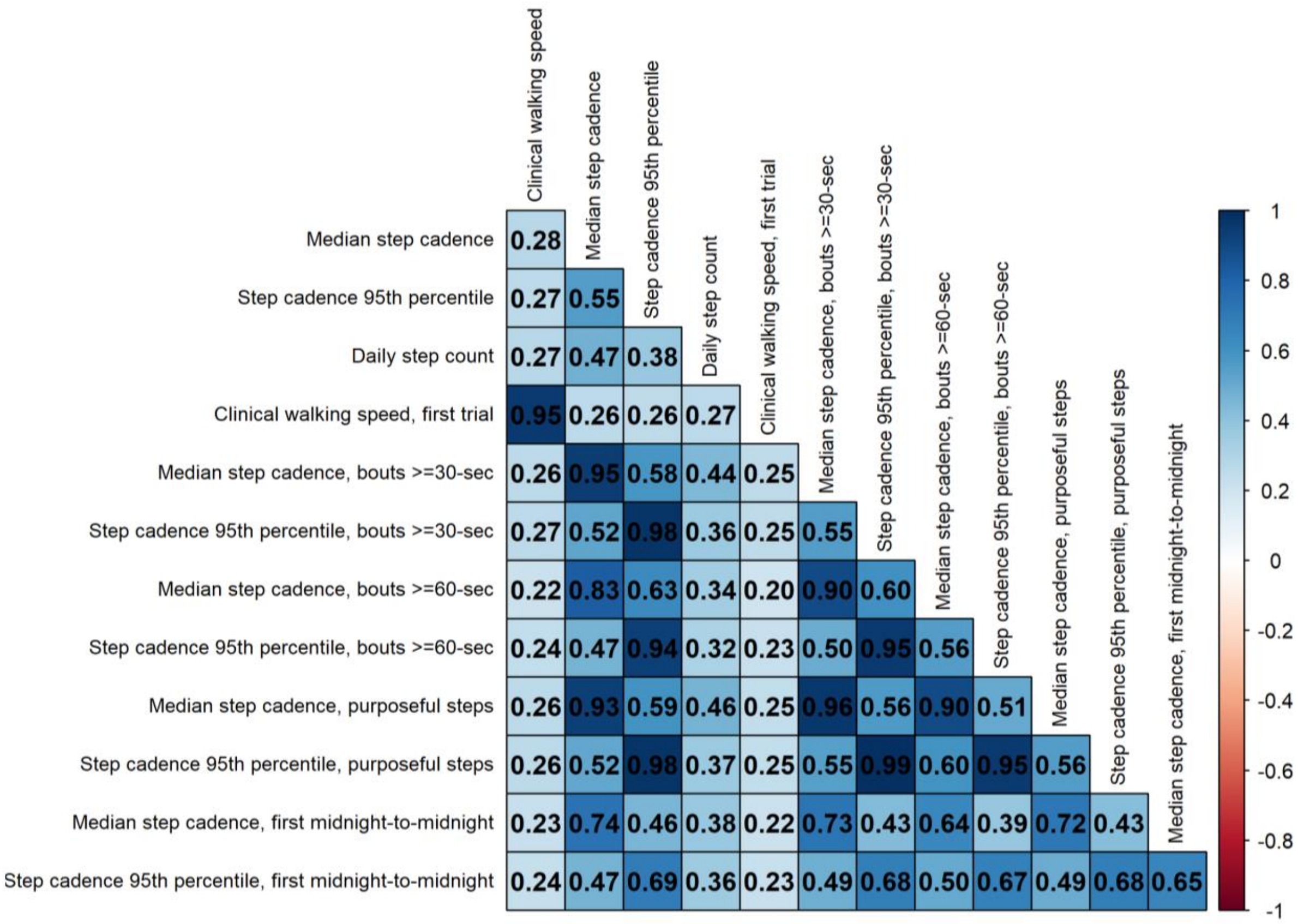

© 2026 Haddadj R et al. *JAMA Network Open*.

eTable 1. Descriptive Characteristics of Whitehall II Participants Comparing Included and Nonincluded in the Analysis

Characteristic	Overall (n=10311)	Included in the analysis: No (n=6244)	Included in the analysis: Yes (n=4067)	p-value
Females	3413 (33.1)	2370 (38.0)	1043 (25.6)	<0.001
Age, years (mean, SD)	69.8 (5.9)	70.8 (6.1)	69.3 (5.7)	<0.001
Education,				<0.001
No academic qualifications	1029 (10.0)	656 (10.5)	373 (9.2)	
Lower secondary school	3870 (37.5)	2583 (41.4)	1287 (31.6)	
Higher secondary school	2745 (26.6)	1610 (25.8)	1135 (27.9)	
University	2099 (20.4)	1157 (18.5)	942 (23.2)	
Higher degree	567 (5.5)	237 (3.8)	330 (8.1)	
Married	4636 (73.8)	1589 (71.6)	3047 (74.9)	<0.001
Non-white ethnicity	1035 (10.1)	729 (11.9)	306 (7.5)	<0.001
Smoking status				<0.001
Never smoker	4295 (47.2)	2307 (45.8)	1988 (48.9)	
Former smoker	4042 (44.4)	2094 (41.6)	1948 (47.9)	
Current smoker	765 (8.4)	634 (12.6)	131 (3.2)	
Alcohol consumption				<0.001
No consumption	2232 (24.5)	1410 (28.0)	822 (20.2)	
1-14 units/week	4874 (53.6)	2583 (51.3)	2291 (56.3)	
>14 units/week	1994 (21.9)	1040 (20.7)	954 (23.5)	
Fruit and vegetable consumption				<0.001
Less than daily	2571 (28.2)	1732 (34.4)	839 (20.6)	
Daily	2399 (26.3)	1532 (30.4)	867 (21.3)	
More than daily	4136 (45.4)	1775 (35.2)	2361 (58.1)	
Body mass index, $m.kg^{-2}$ (mean, SD)	26.5 (4.5)	26.3 (4.6)	26.6 (4.3)	0.004
Hypertension	6372 (61.8)	3966 (63.5)	2406 (59.2)	<0.001
Hyperlipidemia	6195 (68.4)	4115 (82.4)	2080 (51.1)	<0.001
Number of limited ADL (mean, SD)	0.1 (0.6)	0.1 (0.6)	0.2 (0.6)	<0.001
Number of limited IADL (mean, SD)	0.2 (0.6)	0.1 (0.6)	0.2 (0.6)	<0.001
Number of chronic diseases (mean, SD)	0.9 (1.1)	1.1 (1.2)	0.6 (0.8)	<0.001

Data are n (%), unless otherwise indicated. ADL, activity of daily living; IADL, instrumental activity of daily living

© 2026 Haddadj R et al. *JAMA Network Open*.

eTable 2. Descriptive Statistics of Supplementary Walking Parameters Metrics for the Whole Study Sample and Stratified by Sex

		Sex	
	Overall (n=4067)	Female (n=1043)	Male (n=3024)
Clinical walking speed, first trial	1.1 (0.3)	1.0 (0.3)	1.2 (0.3)
Median walking cadence, bout ≥30-sec	77.6 (10.1)	77.5 (10.5)	77.6 (10.0)
95th percentile walking cadence, bout ≥30-sec	113.0 (9.8)	114.7 (9.5)	112.4 (9.8)
Median walking cadence, bout ≥60-sec[a]	83.3 (11.2)	83.7 (11.6)	83.1 (11.1)
95th percentile walking cadence, bout ≥60-sec[a]	114.5 (10.0)	116.5 (9.5)	113.8 (10.1)
Median step cadence, purposeful walking	79.3 (8.9)	79.3 (9.2)	79.3 (8.8)
Step cadence 95th percentile, purposeful walking	113.0 (9.6)	114.7 (9.3)	112.4 (9.6)
Median walking cadence, first midnight-to-midnight[b]	75.5 (12.4)	75.5 (12.4)	75.6 (12.4)
95th percentile walking cadence, first midnight-to-midnight[b]	109.7 (12.3)	111.2 (12.3)	109.2 (12.3)

Data are presented as mean (SD).
[a] Six participants did not have any walking bout ≥60 seconds.
[b] Three participants did not have any complete midnight-to-midnight recording.

© 2026 Haddadj R et al. *JAMA Network Open*.

eTable 3. Association Between Free-Living Walking Cadence and All-Cause Mortality Adjusted for Daily Step Count (N cases/N total: Full Sample, 739/4067; Female, 155/1043; Male, 584/3024)

	**Free-living walking cadence** Hazard ratio per one higher SD (95% CI)	
	**Median walking cadence**	**95th percentile walking cadence**
**Model 1[a]**		
Full sample	0.73 (0.67-0.79)	0.73 (0.67-0.79)
Females	0.80 (0.67-0.95)	0.78 (0.65-0.93)
Males	0.71 (0.64-0.78)	0.73 (0.66-0.80)
**Model 2[b]**		
Full sample	0.85 (0.78-0.93)	0.83 (0.76-0.90)
Females	0.86 (0.72-1.04)	0.81 (0.67-0.99)
Males	0.84 (0.76-0.93)	0.83 (0.75-0.91)
**Model 3[c]**		
Full sample	0.85 (0.78-0.93)	0.83 (0.76-0.91)
Females	0.86 (0.71-1.05)	0.81 (0.67-0.98)
Males	0.85 (0.77-0.94)	0.83 (0.75-0.92)
**Model 4[d]**		
Full sample	0.87 (0.80-0.95)	0.85 (0.79-0.93)
Females	0.87 (0.71-1.05)	0.84 (0.70-1.02)
Males	0.87 (0.79-0.96)	0.85 (0.77-0.94)

Abbreviations: SD, standard deviation; CI, confidence interval.
[a] Adjusted for daily step count.
[b] Additionally adjusted for sex, age, education, marital status, and ethnicity.
[c] Additionally adjusted for smoking status, alcohol consumption, and fruit and vegetable consumption.
[d] Additionally adjusted for body mass index, hypertension, hyperlipidemia, number of activities of daily living limitations, number of instrumental activities of daily living limitations, and number of chronic diseases.

© 2026 Haddadj R et al. *JAMA Network Open*.

eTable 4. Association Between the Walking Parameters and All-Cause Mortality in the Whitehall II Accelerometer Substudy for 0.1 m/s of Clinical Walking Speed and 10 Steps/Minute of Free-Living Walking Cadence

	**Walking parameters** Hazard Ratio (95% CI)		
		**Free-living walking parameters**	
	**Clinical walking speed**	**Median walking cadence**	**95th percentile walking cadence**
**Model 1[a]**			
Full sample	0.83 (0.81-0.86)	0.63 (0.59-0.68)	0.63 (0.58-0.68)
Females	0.79 (0.75-0.84)	0.63 (0.55-0.74)	0.61 (0.52-0.71)
Males	0.81 (0.79-0.84)	0.63 (0.57-0.68)	0.64 (0.58-0.70)
**Model 2[b]**			
Full sample	0.87 (0.85-0.90)	0.78 (0.72-0.84)	0.76 (0.70-0.83)
Females	0.85 (0.80-0.92)	0.75 (0.63-0.89)	0.71 (0.60-0.84)
Males	0.88 (0.85-0.91)	0.78 (0.71-0.86)	0.77 (0.70-0.85)
**Model 3[c]**			
Full sample	0.88 (0.85-0.91)	0.79 (0.73-0.85)	0.77 (0.71-0.84)
Females	0.86 (0.80-0.93)	0.77 (0.64-0.91)	0.73 (0.61-0.86)
Males	0.88 (0.85-0.92)	0.79 (0.72-0.87)	0.78 (0.71-0.86)
**Model 4[d]**			
Full sample	0.90 (0.87-0.93)	0.83 (0.76-0.90)	0.81 (0.75-0.88)
Females	0.89 (0.82-0.96)	0.81 (0.68-0.97)	0.80 (0.67-0.95)
Males	0.91 (0.87-0.94)	0.83 (0.76-0.91)	0.82 (0.74-0.90)

Abbreviations: CI, confidence interval.
[a] Unadjusted.
[b] Adjusted for sex (full sample model only), age, education, marital status, and ethnicity.
[c] Additionally adjusted for smoking status, alcohol consumption, and fruit and vegetable consumption.
[d] Additionally adjusted for body mass index, hypertension, hyperlipidemia, number of activities of daily living limitations, number of instrumental activities of daily living limitations and number of chronic diseases.

© 2026 Haddadj R et al. *JAMA Network Open*.

eTable 5. Association Between Clinical Walking Speed and All-Cause Mortality Including All Participants With Valid Clinical Walking Speed Measurement (N cases/N total: 1079/5492)

	**Hazard ratio (95% CI)**		**C-index (95% CI)**	
	**Analytical sample**	**All available participants**	**Analytical sample**	**All available participants**
Model 1[a]	0.62 (0.58-0.67)	0.58 (0.54-0.61)	0.632 (0.612-0.653)	0.648 (0.631-0.665)
Model 2[b]	0.71 (0.65-0.77)	0.66 (0.61-0.70)	0.732 (0.714-0.749)	0.743 (0.728-0.757)
Model 3[c]	0.72 (0.66-0.78)	0.67 (0.63-0.72)	0.736 (0.718-0.753)	0.747 (0.733-0.762)
Model 4[d]	0.77 (0.71-0.84)	0.74 (0.68-0.79)	0.747 (0.730-0.764)	0.757 (0.743-0.771)

Abbreviations: C-index, Harrel's concordance index; CI, confidence interval.
Hazard ratio is reported per one higher standard deviation.
[a] Adjusted for daily step count.
[b] Additionally adjusted for sex, age, education, marital status, and ethnicity.
[c] Additionally adjusted for smoking status, alcohol consumption, and fruit and vegetable consumption.
[d] Additionally adjusted for body mass index, hypertension, hyperlipidemia, number of activities of daily living limitations, number of instrumental activities of daily living limitations, and number of chronic diseases.

© 2026 Haddadj R et al. *JAMA Network Open*.

eFigure 5. Calibration of the Walking Parameters for the 11-Year Risk of All-Cause Mortality

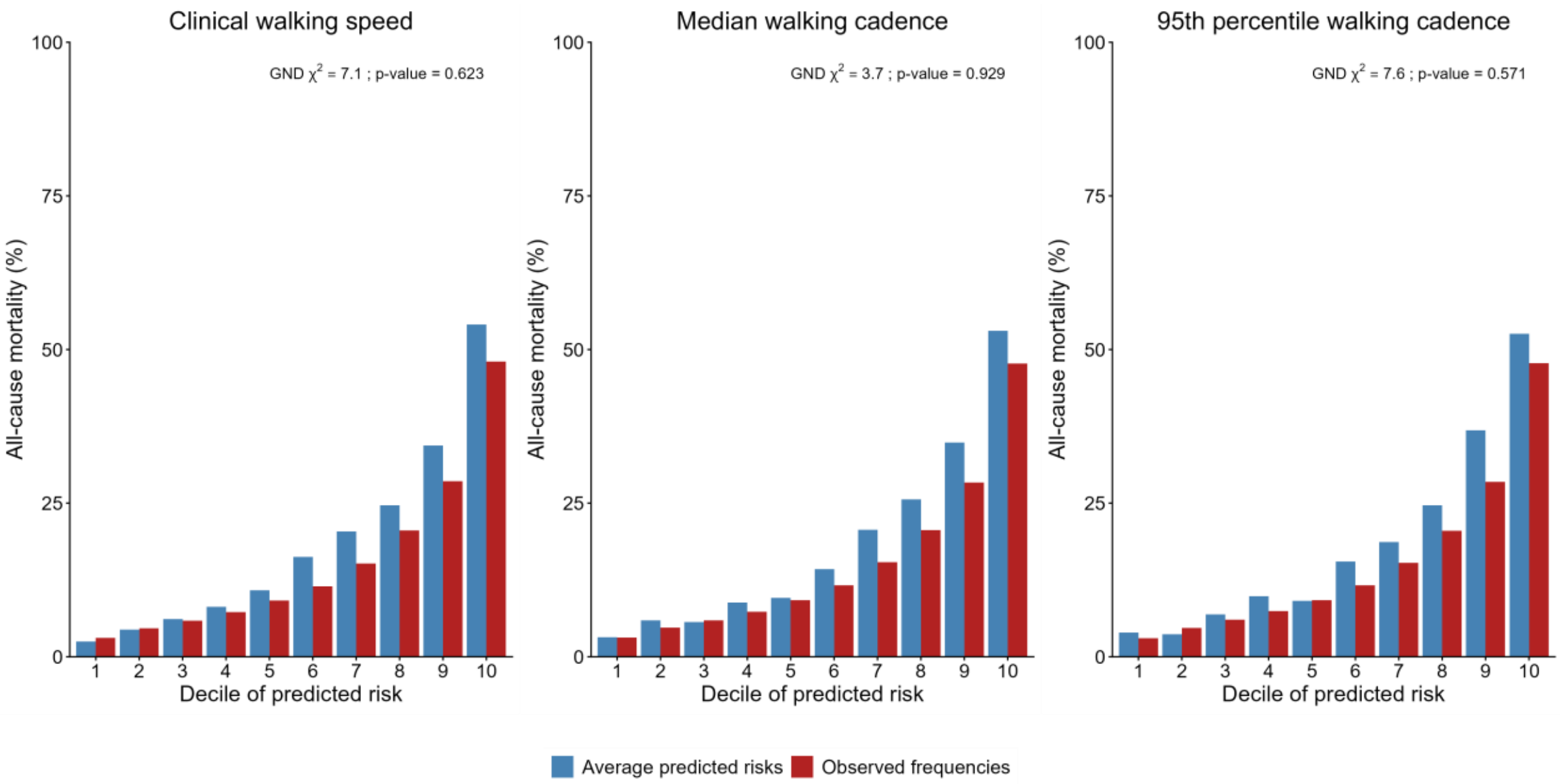

Abbreviations: GND, Greenwood-Nam-D'Agostino test.

Models are adjusted for sex, age, education, marital status, ethnicity, smoking status, alcohol consumption, fruit and vegetable consumption, body mass index, hypertension, hyperlipidemia, number of activities of daily living limitations, number of instrumental activities of daily living limitations, and number of chronic diseases.

© 2026 Haddadj R et al. *JAMA Network Open*.

eFigure 6. Hazard Ratios (A) and Discriminative Ability (B) of Walking Parameters for All-Cause Mortality Risk by Age (N Cases/N Total: in Younger Group, age <68.4 Years, 166/2033; in Older Group, Age ≥68.4 Years, 573/2034)

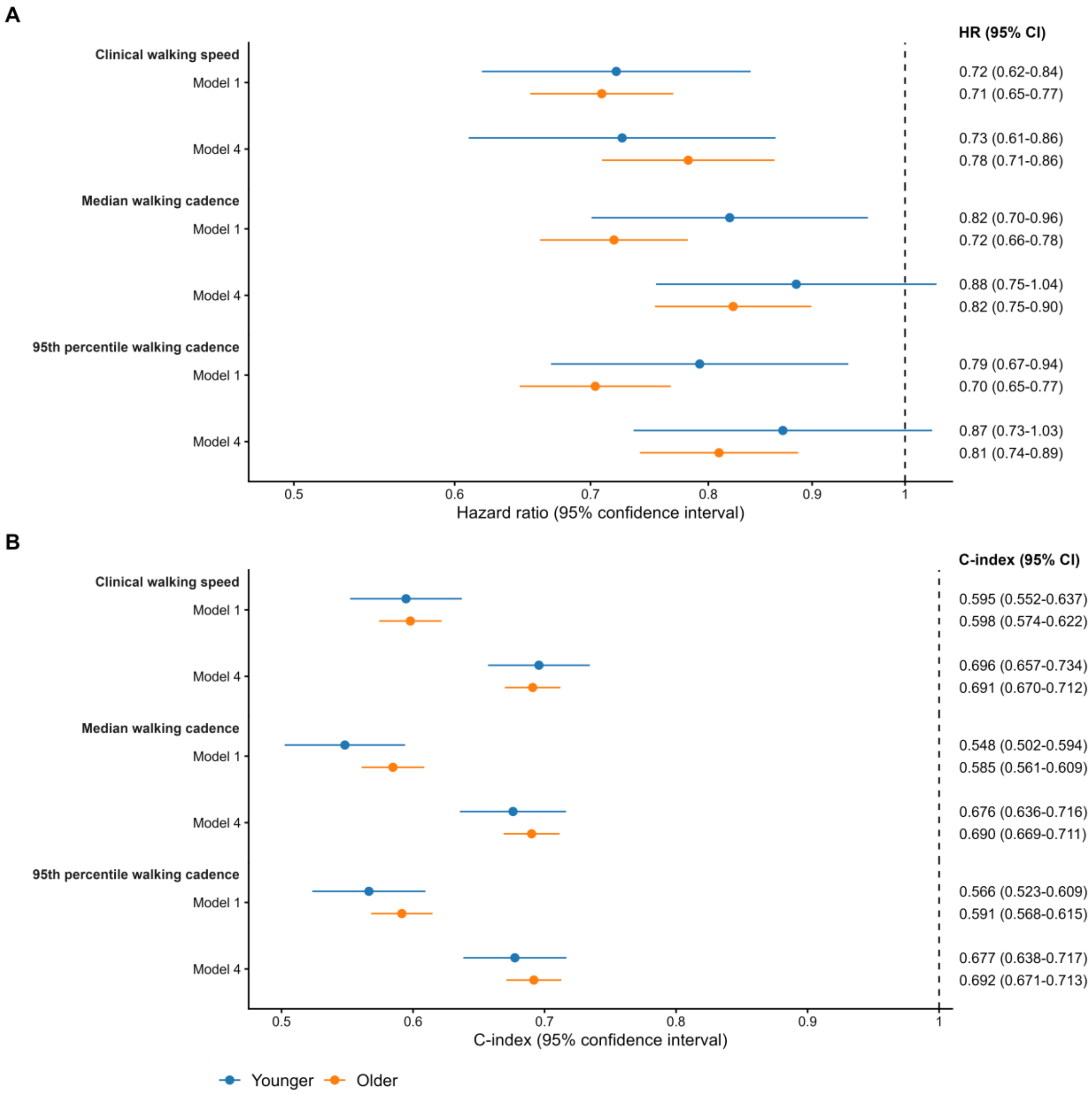

The x-axis for panel A is a log scale.
Abbreviations: HR, hazard ratio; CI, confidence interval; C-index, Harrel's concordance-index.
Model 1 is unadjusted.
Model 4 (named as in the main analysis for consistency) is adjusted for sex, age, education, marital status, ethnicity, smoking status, alcohol consumption, fruit and vegetable consumption, body mass index, hypertension, hyperlipidemia, number of activities of daily living limitations, number of instrumental activities of daily living limitations, and number of chronic diseases.
There was no evidence of interaction between walking parameters and age.
There was no significant difference in discriminative ability between subgroups.

© 2026 Haddadj R et al. *JAMA Network Open*.

eFigure 7. Hazard Ratios (A) and Discriminative Ability (B) of Walking Parameters for All-Cause Mortality Risk by Body Mass Index (N Cases/N Total: in Those with BMI <25kg/m², 281/1570; in Those with BMI ≥25 kg/m², 458/2497)

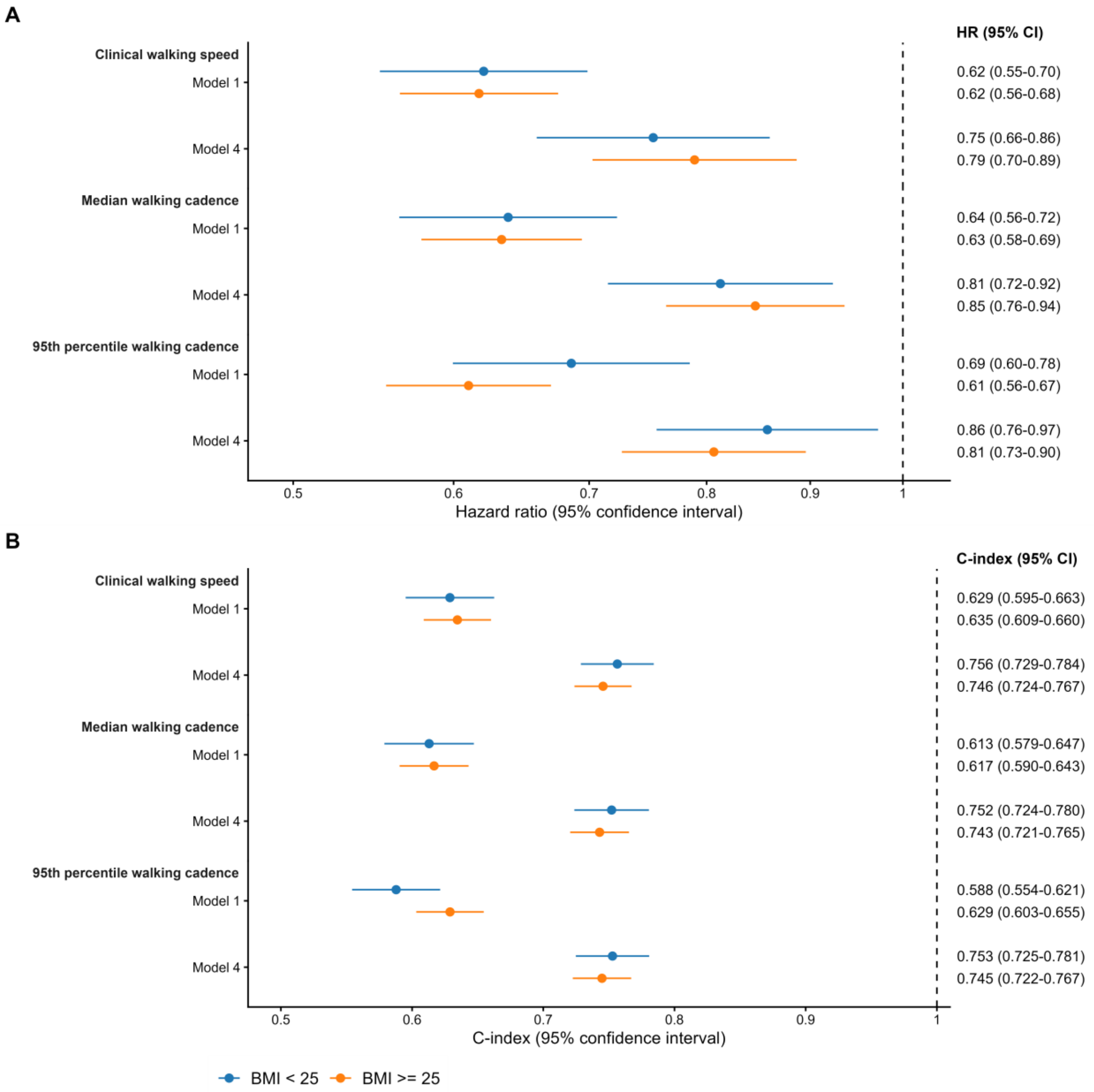

The x-axis for panel A is a log scale.
Abbreviations: HR, hazard ratio; CI, confidence interval; C-index, Harrel's concordance-index.
Model 1 is unadjusted.
Model 4 (named as in the main analysis for consistency) is adjusted for sex, age, education, marital status, ethnicity, smoking status, alcohol consumption, fruit and vegetable consumption, body mass index, hypertension, hyperlipidemia, number of activities of daily living limitations, number of instrumental activities of daily living limitations, and number of chronic diseases.
There was no evidence for interaction between walking parameters and BMI.
There was no significant difference in discriminative ability between subgroups.

© 2026 Haddadj R et al. *JAMA Network Open*.

eFigure 8. Hazard Ratios (A) and Discriminative Ability (B) of Walking Parameters for All-Cause Mortality Risk by Prevalence of at Least 1 Chronic Disease at Baseline (N Cases/N Total: in Those With no Chronic Disease, 306/2278; in Those With at Least 1 Chronic Disease, 433/1789)

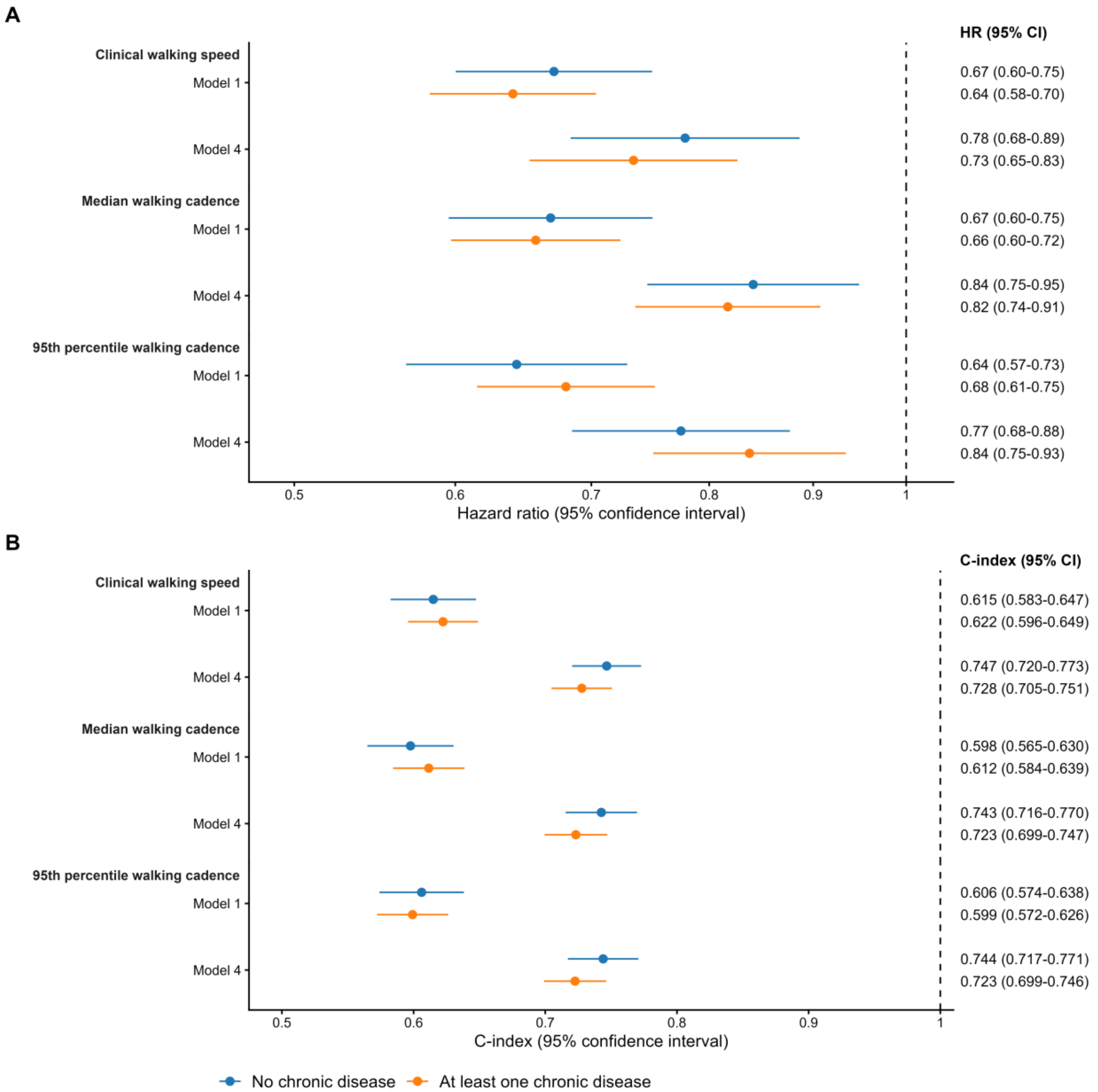

The x-axis for panel A is a log scale.
Abbreviations: HR, hazard ratio; CI, confidence interval; C-index, Harrel's concordance-index.
Model 1 is unadjusted.
Model 4 (named as in the main analysis for consistency) is adjusted for sex, age, education, marital status, ethnicity, smoking status, alcohol consumption, fruit and vegetable consumption, body mass index, hypertension, hyperlipidemia, number of activities of daily living limitations, and number of instrumental activities of daily living limitations.
There was no evidence of interaction between walking parameters and number of chronic diseases.
There was no significant difference in discriminative ability between subgroups.

© 2026 Haddadj R et al. *JAMA Network Open*.

eFigure 9. Hazard Ratios (A) and Discriminative Ability (B) of Walking Parameters for All-Cause Mortality Excluding Participants that Died During the First 2 Years of Follow-Up (N Cases/N Total = 691/4019)

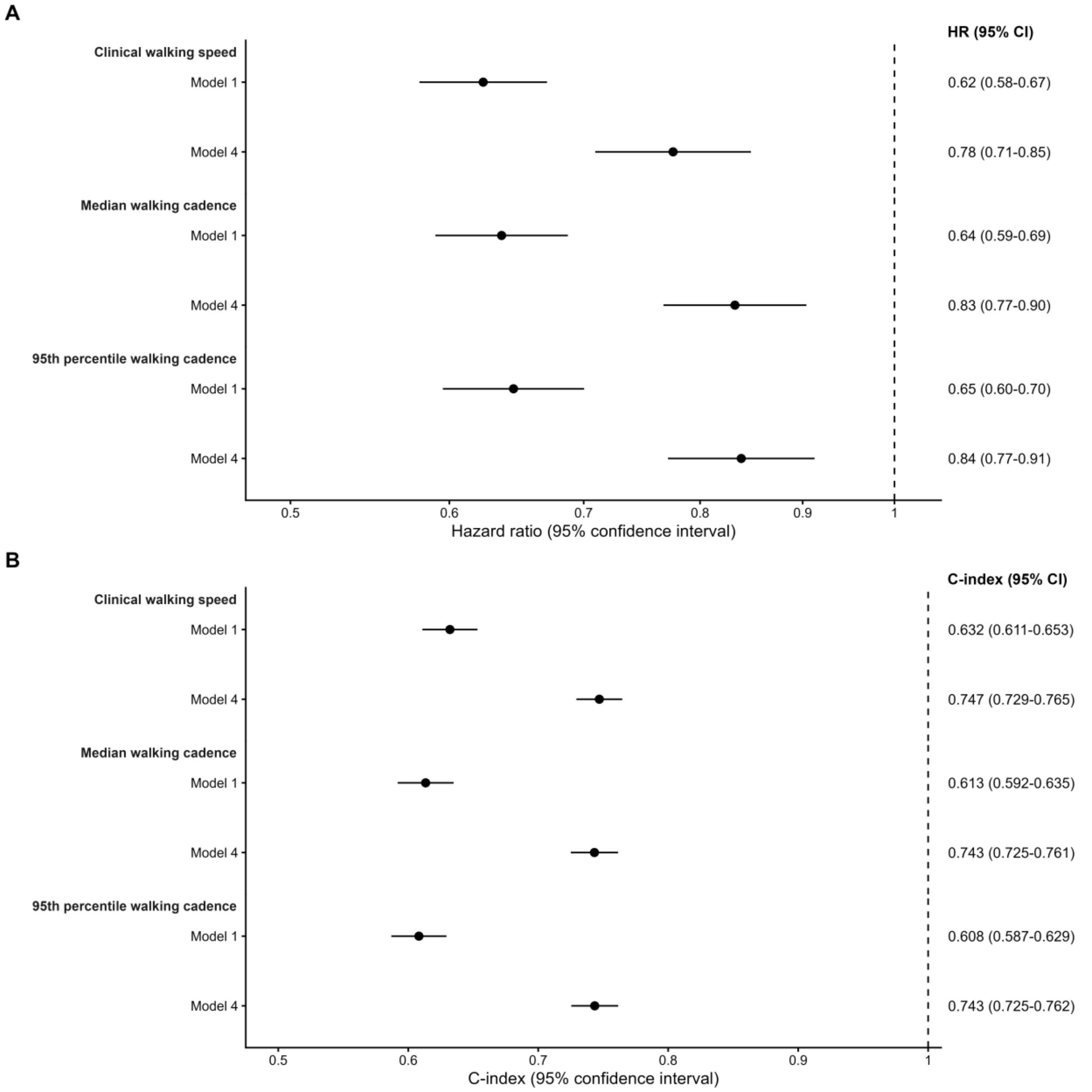

The x-axis for panel A is a log scale.
Abbreviations: HR, hazard ratio; CI, confidence interval; C-index, Harrel's concordance-index.
Model 1 is unadjusted.
Model 4 (named as in the main analysis for consistency) is adjusted for sex, age, education, marital status, ethnicity, smoking status, alcohol consumption, fruit and vegetable consumption, body mass index, hypertension, hyperlipidemia, number of activities of daily living limitations, number of instrumental activities of daily living limitations, and number of chronic diseases.

© 2026 Haddadj R et al. *JAMA Network Open*.

eFigure 10. Hazard Ratios (A) and Discriminative Ability (B) of Walking Parameters for All-Cause Mortality Risk Including Participants With ≥7 Valid Days of Accelerometer Wear (N Cases/N Total = 708/3905)

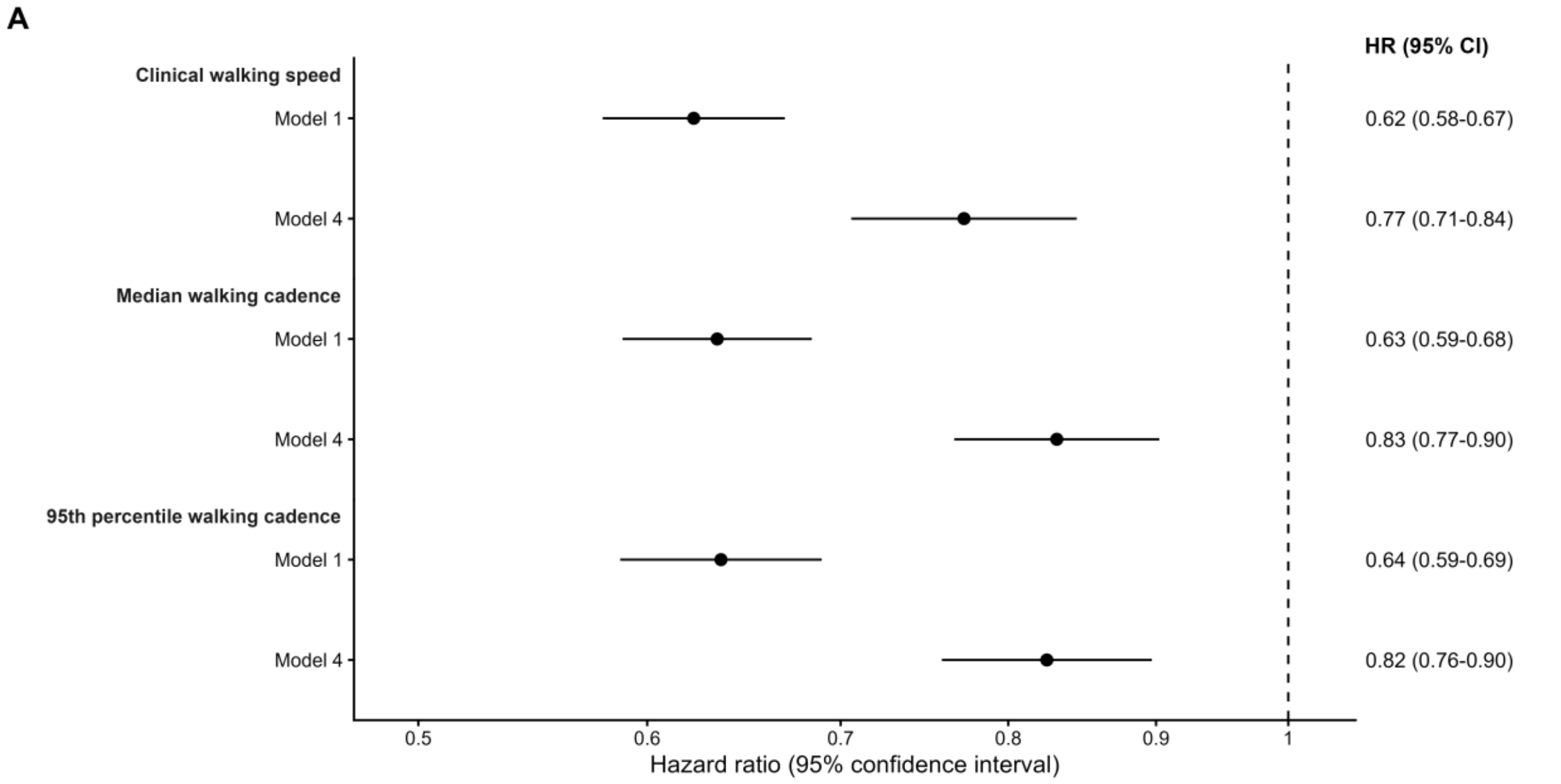

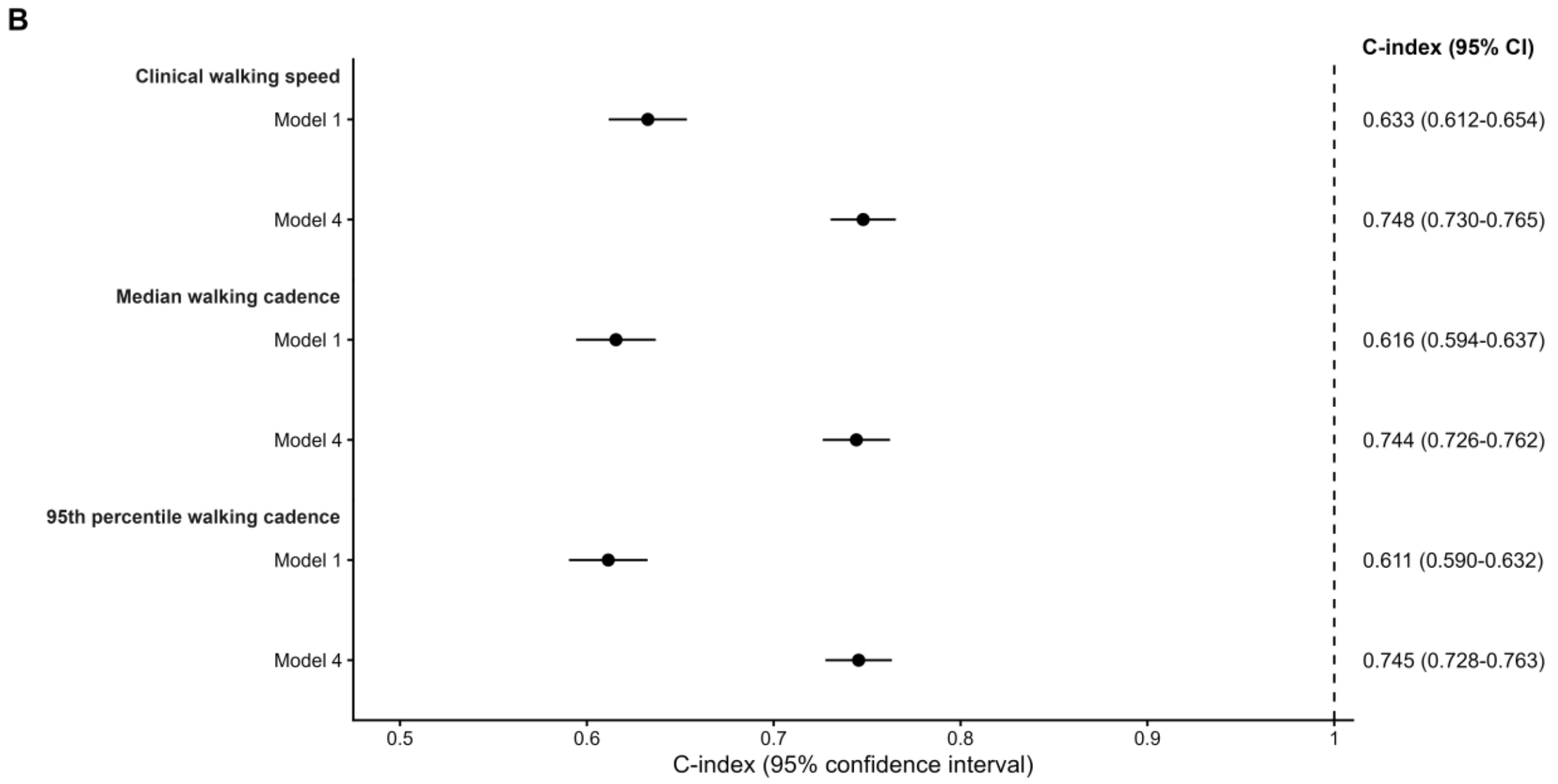

The x-axis for panel A is a log scale.
Abbreviations: HR, hazard ratio; CI, confidence interval; C-index, Harrel's concordance-index.
Model 1 is unadjusted.
Model 4 (named as in the main analysis for consistency) is adjusted for sex, age, education, marital status, ethnicity, smoking status, alcohol consumption, fruit and vegetable consumption, body mass index, hypertension, hyperlipidemia, number of activities of daily living limitations, number of instrumental activities of daily living limitations, and number of chronic diseases.

© 2026 Haddadj R et al. *JAMA Network Open*.

eFigure 11. Hazard Ratios (A) and Discriminative Ability (B) of Walking Parameters for All-Cause Mortality Risk With Walking Cadence Derived From Walking Bouts ≥30 Seconds (N Cases/N Total = 739/4067)

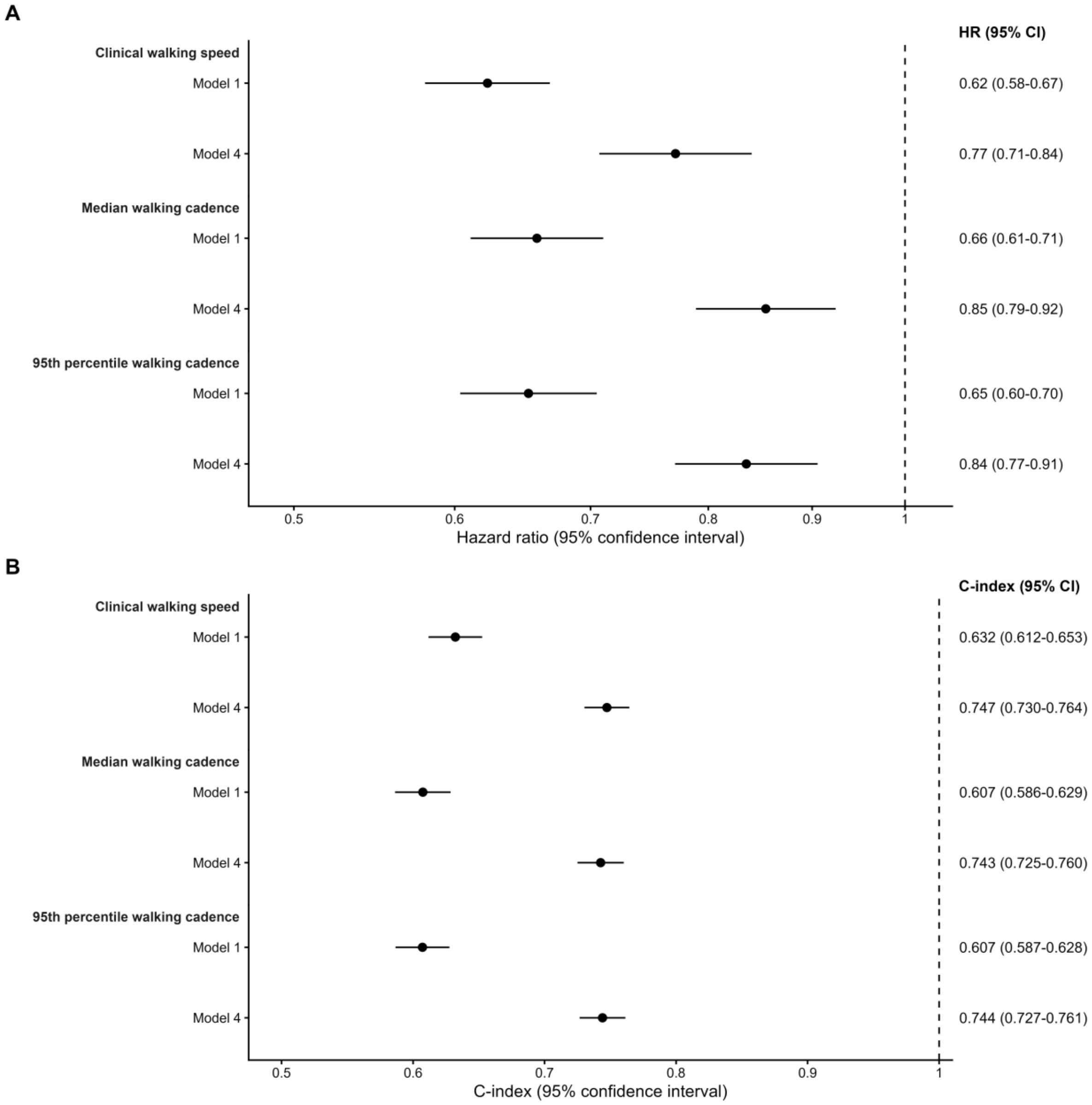

The x-axis for panel A is a log scale.
Abbreviations: HR, hazard ratio; CI, confidence interval; C-index, Harrel's concordance-index.
Model 1 is unadjusted.
Model 4 (named as in the main analysis for consistency) is adjusted for sex, age, education, marital status, ethnicity, smoking status, alcohol consumption, fruit and vegetable consumption, body mass index, hypertension, hyperlipidemia, number of activities of daily living limitations, number of instrumental activities of daily living limitations, and number of chronic diseases.

eFigure 12. Hazard Ratios (A) and Discriminative Ability (B) of Walking Parameters for All-Cause Mortality Risk With Walking Cadence Derived From Walking Bouts ≥60 Seconds (N Cases/N Total = 736/4061)

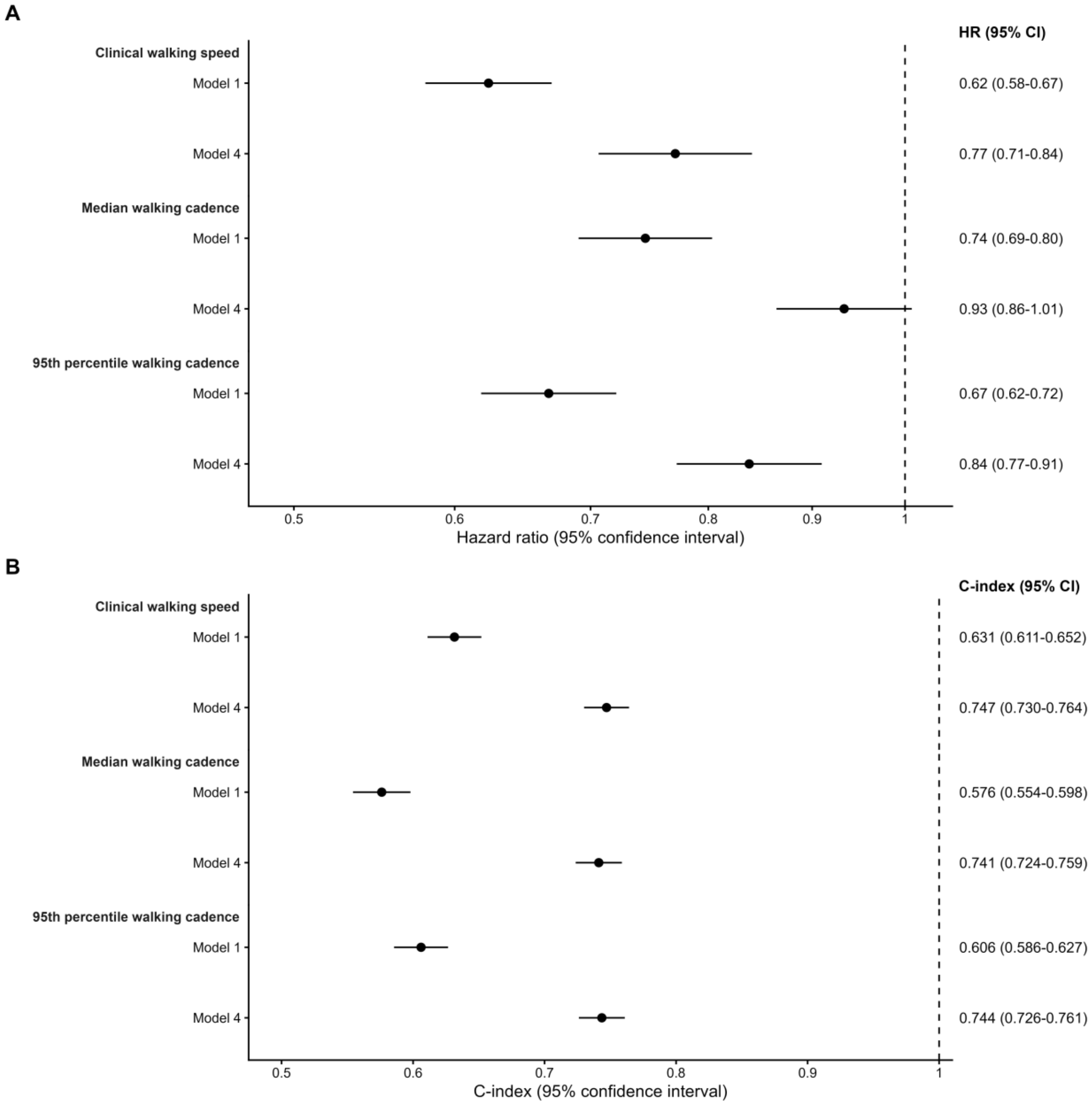

The x-axis for panel A is a log scale.

Abbreviations: HR, hazard ratio; CI, confidence interval; C-index, Harrel's concordance-index.

Model 1 is unadjusted.

Model 4 (named as in the main analysis for consistency) is adjusted for sex, age, education, marital status, ethnicity, smoking status, alcohol consumption, fruit and vegetable consumption, body mass index, hypertension, hyperlipidemia, number of activities of daily living limitations, number of instrumental activities of daily living limitations, and number of chronic diseases.

© 2026 Haddadj R et al. *JAMA Network Open*.

eFigure 13. Hazard Ratios (A) and Discriminative Ability (B) of Walking Parameters for All-Cause Mortality Risk With Walking Cadence Derived From Purposeful Walking (≥40 Steps/Minute Over a 10-Second Window) (N cases/N total = 739/4067)

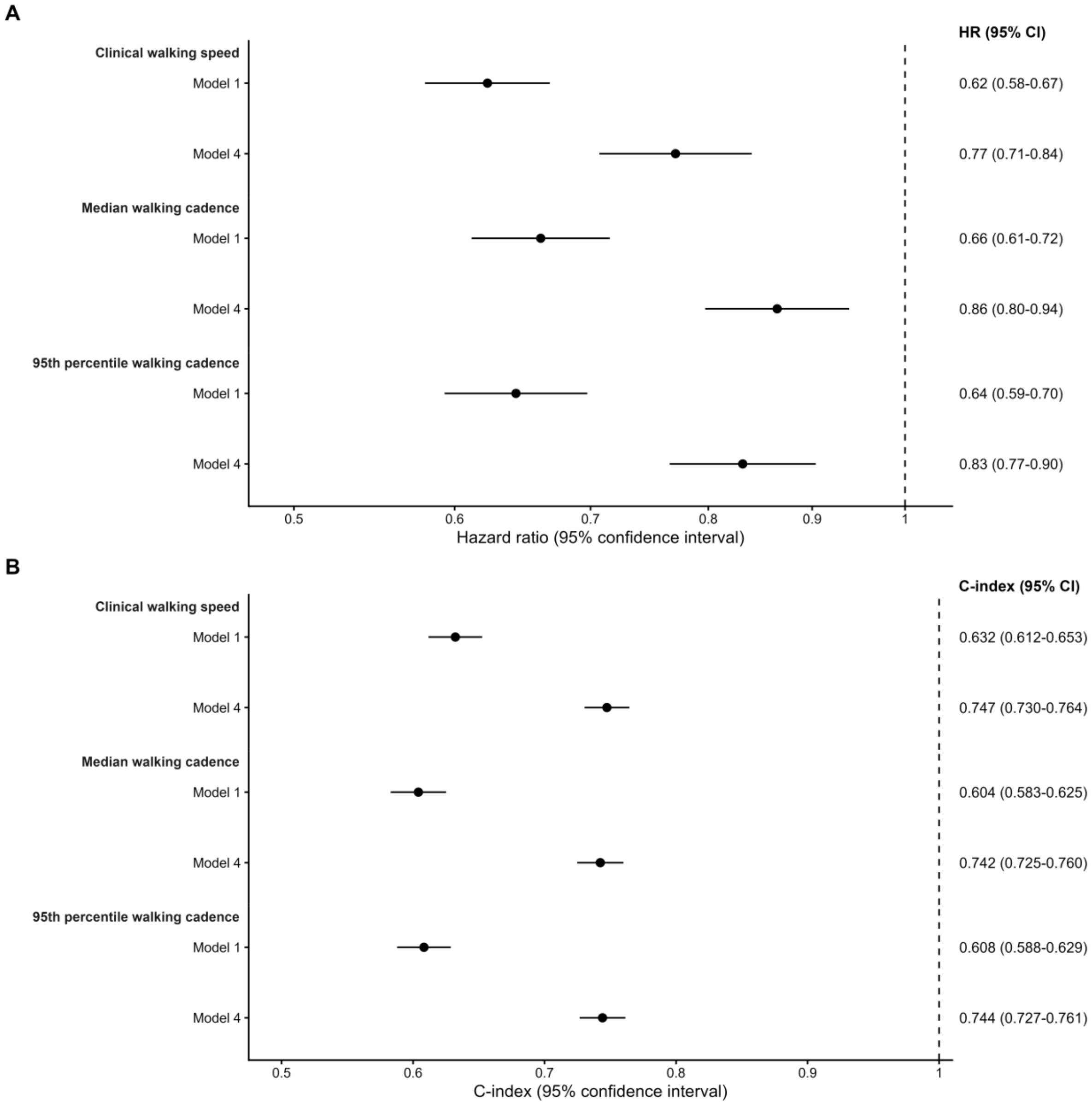

The x-axis for panel A is a log scale.
Abbreviations: HR, hazard ratio; CI, confidence interval; C-index, Harrel's concordance-index.
Model 1 is unadjusted.
Model 4 (named as in the main analysis for consistency) is adjusted for sex, age, education, marital status, ethnicity, smoking status, alcohol consumption, fruit and vegetable consumption, body mass index, hypertension, hyperlipidemia, number of activities of daily living limitations, number of instrumental activities of daily living limitations, and number of chronic diseases.

© 2026 Haddadj R et al. *JAMA Network Open*.

eFigure 14. Hazard Ratios (A) and Discriminative Ability (B) of Walking Parameters for All-Cause Mortality Risk With Walking Cadence Derived From the First Complete 24-Hour (N Cases/N Total = 737/4064)

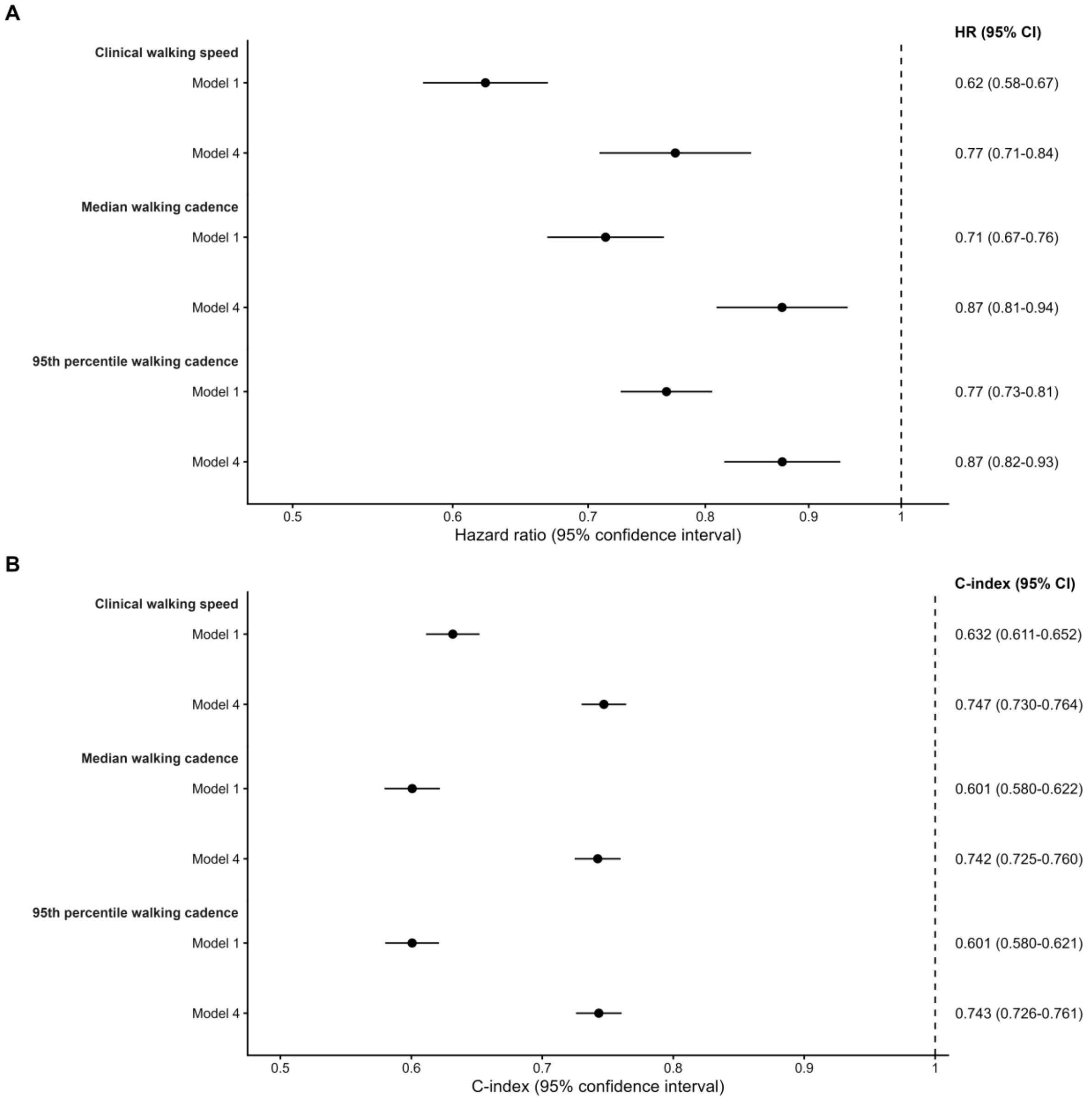

The x-axis for panel A is a log scale.

Abbreviations: HR, hazard ratio; CI, confidence interval; C-index, Harrel's concordance-index.

Model 1 is unadjusted.

Model 4 (named as in the main analysis for consistency) is adjusted for sex, age, education, marital status, ethnicity, smoking status, alcohol consumption, fruit and vegetable consumption, body mass index, hypertension, hyperlipidemia, number of activities of daily living limitations, number of instrumental activities of daily living limitations, and number of chronic diseases.

© 2026 Haddadj R et al. *JAMA Network Open*.

eFigure 15. Hazard Ratios (A) and Discriminative Ability (B) of Walking Parameters for All-Cause Mortality With Clinical Walking Speed Derived From the First Trial of the Walking Test (N Cases/N Total = 739/4067)

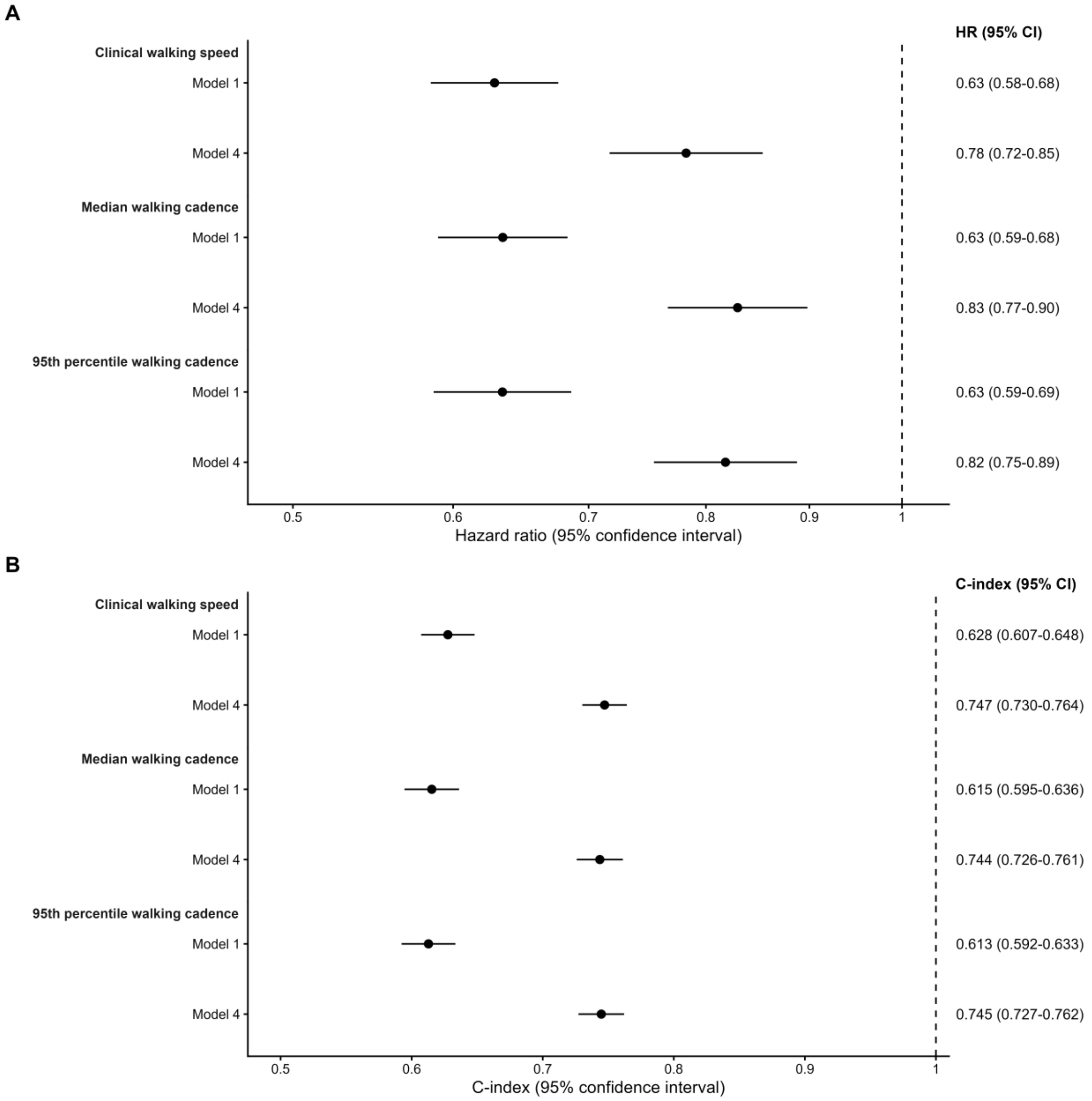

The x-axis for panel A is a log scale.
Abbreviations: HR, hazard ratio; CI, confidence interval; C-index, Harrel's concordance-index.
Model 1 is unadjusted.
Model 4 (named as in the main analysis for consistency) is adjusted for sex, age, education, marital status, ethnicity, smoking status, alcohol consumption, fruit and vegetable consumption, body mass index, hypertension, hyperlipidemia, number of activities of daily living limitations, number of instrumental activities of daily living limitations, and number of chronic diseases.

© 2026 Haddadj R et al. *JAMA Network Open*.
