# Supplementary material for: Clinical Walking Speed, Free-Living Walking Cadence, and All-Cause Mortality
Source: JAMA Netw Open. 2026 Oct 23;9(10):e2640122. doi: 10.1001/jamanetworkopen.2026.40122 (PMC13695056; doi:10.1001/jamanetworkopen.2026.40122)
Supplement: Supplementary file 2 [file jamanetwopen-e2640122-s002.pdf]

Data Sharing Statement

Haddadj. Clinical Walking Speed, Free-Living Walking Cadence, and All-Cause Mortality. *JAMA Netw Open*. Published October 23, 2026. doi:10.1001/jamanetworkopen.2026.40122

Data
Data available: No

Additional Information
Explanation for why data not available: Data cannot be made publicly available because of ethics and IRB restrictions. However, a data sharing portal allows access to data to undertake analyses within the secure portal in WII https://portal.dementiasplatform.uk/.
